# Loss of SETDB1 decompacts the inactive X chromosome in part through reactivation of an enhancer in the *IL1RAPL1* gene

**DOI:** 10.1186/s13072-018-0218-9

**Published:** 2018-08-13

**Authors:** Zhuo Sun, Brian P. Chadwick

**Affiliations:** Department of Biological Science, Florida State University, 319 Stadium Drive, King 3076, Tallahassee, FL 32306-4295 USA

**Keywords:** X chromosome inactivation, Heterochromatin, SETDB1, IL1RAPL1, Enhancer, Inactive X chromosome, Euchromatin

## Abstract

**Background:**

The product of dosage compensation in female mammals is the inactive X chromosome (Xi). Xi facultative heterochromatin is organized into two different types, one of which is defined by histone H3 trimethylated at lysine 9 (H3K9me3). The rationale for this study was to assess SET domain bifurcated 1 (SETDB1) as a candidate for maintaining this repressive modification at the human Xi.

**Results:**

Here, we show that loss of SETDB1 does not result in large-scale H3K9me3 changes at the Xi, but unexpectedly we observed striking decompaction of the Xi territory. Close examination revealed a 0.5 Mb region of the Xi that transitioned from H3K9me3 heterochromatin to euchromatin within the 3′ end of the *IL1RAPL1* gene that is part of a common chromosome fragile site that is frequently deleted or rearranged in patients afflicted with intellectual disability and other neurological ailments. Centrally located within this interval is a powerful enhancer adjacent to an ERVL-MaLR element. In the absence of SETDB1, the enhancer is reactivated on the Xi coupled with bidirectional transcription from the ERVL-MaLR element. Xa deletion of the enhancer/ERVL-MaLR resulted in loss of full-length IL1RAPL1 transcript in cis, coupled with trans decompaction of the Xi chromosome territory, whereas Xi deletion increased detection of full-length IL1RAPL1 transcript in trans, but did not impact Xi compaction.

**Conclusions:**

These data support a critical role for SETDB1 in maintaining the ERVL-MaLR element and adjacent enhancer in the 3′ end of the IL1RAPL1 gene in a silent state to facilitate Xi compaction.

**Electronic supplementary material:**

The online version of this article (10.1186/s13072-018-0218-9) contains supplementary material, which is available to authorized users.

## Background

The XX/XY chromosome mode of sex determination necessitates the need to compensate levels of X-linked gene expression between the sexes. In mammals, this is achieved through the process of X chromosome inactivation (XCI), whereby one of the two X chromosomes in females is rendered transcriptional silent early in development [1]. Gene silencing at the inactive X chromosome (Xi) is achieved by repackaging the chromosome into facultative heterochromatin, which includes acquisition of repressive histone marks such as histone H3 methylated at lysine 9 and trimethylated at lysine 27 (H3K27me3) [2–5]. Histone H3 trimethylated at lysine 9 (H3K9me3) and H3K27me3 occupy distinct regions of the Xi [6–8], with H3K27me3 bands associating with gene-rich regions of the chromosome and H3K9me3 predominantly occupying gene-poor regions [7, 9]. H3K27me3 is catalyzed by the histone methyltransferase (HMTase) enhancer of Zeste 2 (EZH2) [10–13], which applies the mark to chromatin of the Xi early during XCI [4, 5]. In contrast, while H3K9me3 has been reported at the Xi [6–8], which HMTase is responsible for the maintenance of this modification at the Xi is uncertain [14, 15]. Recent data suggest that SET domain bifurcated 1 (SETDB1), an H3-K9 HMTase with highest activity for trimethylation of lysine 9 [16, 17], is involved in maintaining gene silencing on the mouse Xi [18], and while it is important for establishing H3K9me3 on the mouse Xi [7] and is enriched on the vole Xi in trophoblast stem cells [19], a central role for this protein in maintaining this mark post-XCI is less certain [7]. SETDB1 is important for gene and repeat silencing during development [20], and a role for this protein in maintaining H3K9me3 at the human Xi has not been explored. Therefore, we sought to further investigate the relationship between SETDB1 and the human Xi.

By generating SETDB1 knockouts (KO) in human cells, we reveal that while SETDB1 is not required to maintain chromosome-wide H3K9me3 at the human Xi, its loss results in decompaction of the Xi chromosome territory. The most obvious chromatin change in the Xi corresponds to a 0.5-Mb interval embedded within the 3′ end of the large interleukin-1 receptor accessory protein-like 1 (*IL1RAPL1*) gene that transitioned from a H3K9me3-defined region of heterochromatin to euchromatin. Centrally located within this interval we identified a powerful enhancer element adjacent to an ERVL-MaLR that is reactivated from the Xi. Through characterization of these elements and their deletion from either the Xa or Xi, we reveal complex cis and trans effects that influence IL1RAPL1 transcription and chromosome compaction.

## Results

### SETDB1 loss results in decompaction of the territory of the Xi

**Fig. 1:**
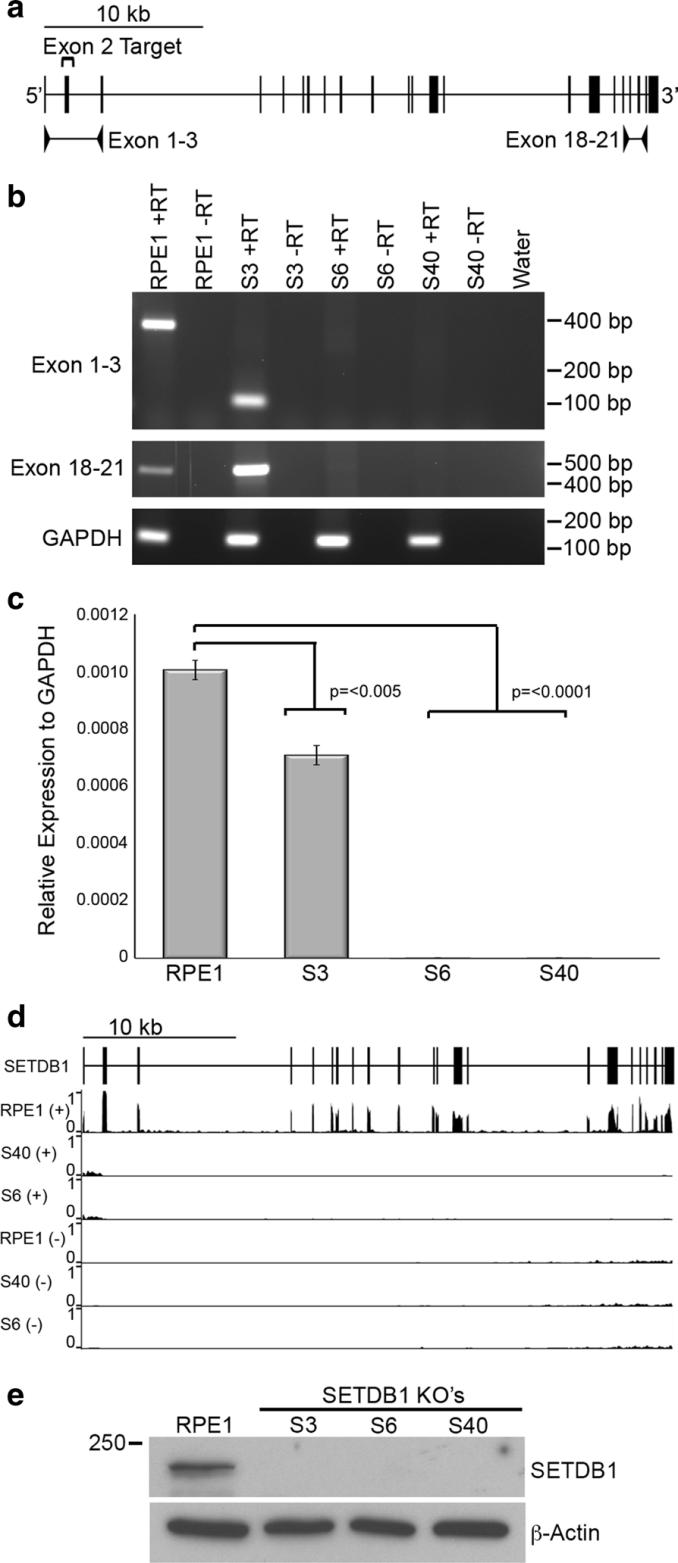
Promoter-trap targeting of exon-2 results in loss of SETDB1 protein. **a** Schematic map of the *SETDB1* locus corresponding to Chr1: 150,926,412–150,964,744 bp, hg38. Vertical lines correspond to the 22 exons of *SETDB1*. Exon-2 that was the target for TALEN cutting is indicated as the location of primers used for RT-PCR between exons 1–3 and 18–21. **b** Ethidium bromide-stained agarose gel image of RT-PCR data for the samples indicated. Annotation of +RT indicates cDNA prepared with reverse transcriptase, whereas − RT was without, serving as a control for genomic DNA carryover. Size in base pairs is indicated to the right. **c** qRT-PCR data for SETDB1 shown relative to GAPDH expression (*y*-axis). Bars correspond to combined data from three biological replicates, each performed as three technical replicates. Error bars show standard error of the mean, and *p* values were derived from performing unpaired *t* tests. **d** *SETDB1* locus RNA-Seq data for RPE1 and SETDB1 KO clones showing forward (+)- and reverse (−)-strand profiles for the same interval as above in part-a. **e** Western blot showing absence of SETDB1 protein in three independent knockout clones. Molecular weight in kD is indicated on the left. The loading levels are indicated by the β-actin signal

**Fig. 2:**
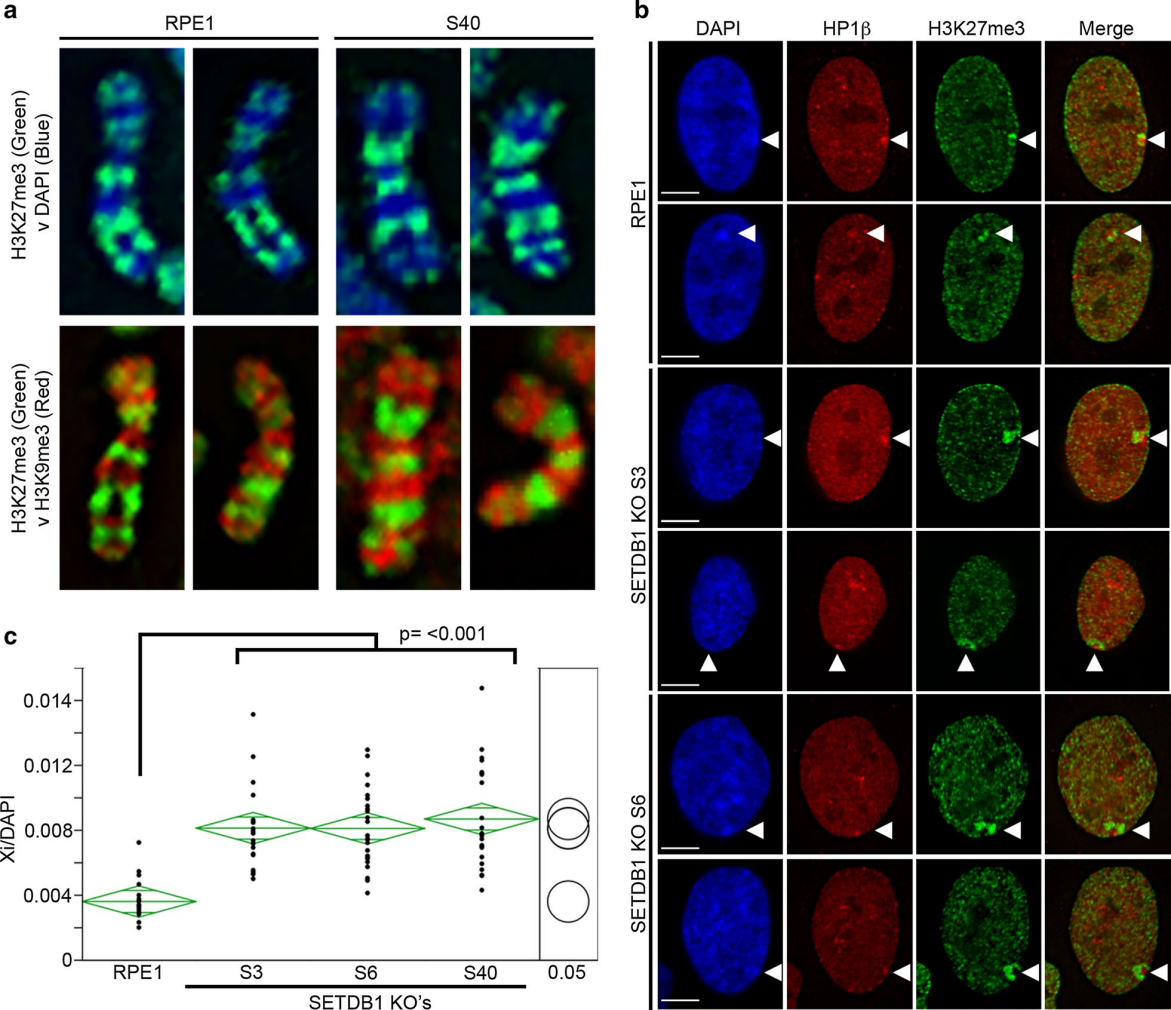
SETDB1 loss is coupled with expansion of the Xi territory. **a** Distribution of heterochromatin bands on the Xi in parental RPE1 cells and SETDB1-targeted clone S40. The top panels show indirect immunofluorescence patterns of H3K27me3 (Green) merged with DAPI (blue), whereas lower panels show representative examples of indirect immunofluorescence pattern of H3K27me3 (green) merged with the pattern of H3K9me3 detected by direct immunofluorescence (red). **b** Indirect immunofluorescence showing DAPI (blue), the distribution of HP1β (Red), H3K27me3 (green), and a merge of the two in RPE1 and two SETDB1 KO clones. White arrow heads indicate the location of the Xi. **c** Volume measurements of the Xi in RPE1 compared to the three KO clones displayed as oneway ANOVA. P-values calculated using Tukey–Kramer honest significant difference. Xi/DAPI volumes are indicated on the *y*-axis. Each black dot indicates a measurement made for individual nuclei in each sample. The green diamonds show the mean (central horizontal line) and 95% confidence interval between the apexes of the diamond. The width of the diamond is proportional to the sample size with wider diamonds indicating more measured nuclei. Circles on the far right show all pairs Tukey–Kramer 0.05 *p* value spread with significance presented by the angle of intersection between circles

In order to knock out SETDB1, we used the fast ligation-based automatable solid-phase high-throughput system (FLASH) to assemble a pair of previously reported active transcription activator-like effector nuclease (TALENs) that target exon-2 (Fig. 1a) of the SETDB1 locus [21]. These were introduced into hTERT-RPE1 (RPE1) cells along with a promoterless neomycin trap targeting construct [22] to act as a template for homology-mediated repair (Additional file 1). Numerous independent targeted clones were isolated (Additional file 2), and three were selected for further investigation. Two clones (S6 and S40) had exchanged exon-2 with the neomycin cassette at both alleles. The promoter trap construct is designed to terminate transcription immediately after transcribing through the cassette. Therefore, we would expect not to be able to detect SETDB1 RNA beyond exon-1 for these clones, which was indeed the case with the lack of product by reverse transcription PCR (RT-PCR) (Fig. 1b) and quantitative RT-PCR (qRT-PCR) (Fig. 1c). Furthermore, massively parallel sequencing of RNA (RNA-Seq) shows a lack of signal at and beyond exon-2 (Fig. 1d) confirming the lack of SETDB1 transcript in these clones. The third clone (S3) had incorporated the neomycin cassette at one allele, whereas the second allele was disrupted by non-homologous end joining. As such, we would expect transcript lacking exon-2 (and the translation initiation codon) to be generated from this allele. RT-PCR clearly detects transcript without exon-2 (Fig. 1b) that exists at significantly lower levels than parental cells (Fig. 1c). Western blot analysis using an antibody raised against the C-terminus of the protein failed to detect SETDB1 protein in any of the three clones, validating each as SETDB1-KOs (Fig. 1e). Initially we looked to see whether loss of SETDB1 impacted facultative heterochromatin bands along the length of the Xi at metaphase. However, the typical alternating banding pattern of H3K9me3 and H3K27me3 at the RPE1 Xi [6] was not obviously impacted (Fig. 2a). Next, we examined the organization of the Xi at interphase. RPE1 and SETDB1-KO clones were stained for H3K27me3 and heterochromatin protein 1 beta (HP1β) as a marker of the H3K9me3 territory. While both territories of the bipartite structure could be detected [6], we found that in SETDB1-KO clones the H3K27me3 territory was very obvious and substantially larger than that of parental RPE1 cells (Fig. 2b) in the absence of any increase in overall nuclear volume (Additional file 3) or change to H3K27me3 banding at the Xi (Fig. 2a). Volume measurements of H3K27me3 at the Xi confirmed that the Xi volume had significantly increased in SETDB1-KO clones (Fig. 2c).

### Loss of SETDB1 results in gain of euchromatin within the 3′ end of the *IL1RAPL1* gene on the Xi

**Fig. 3:**
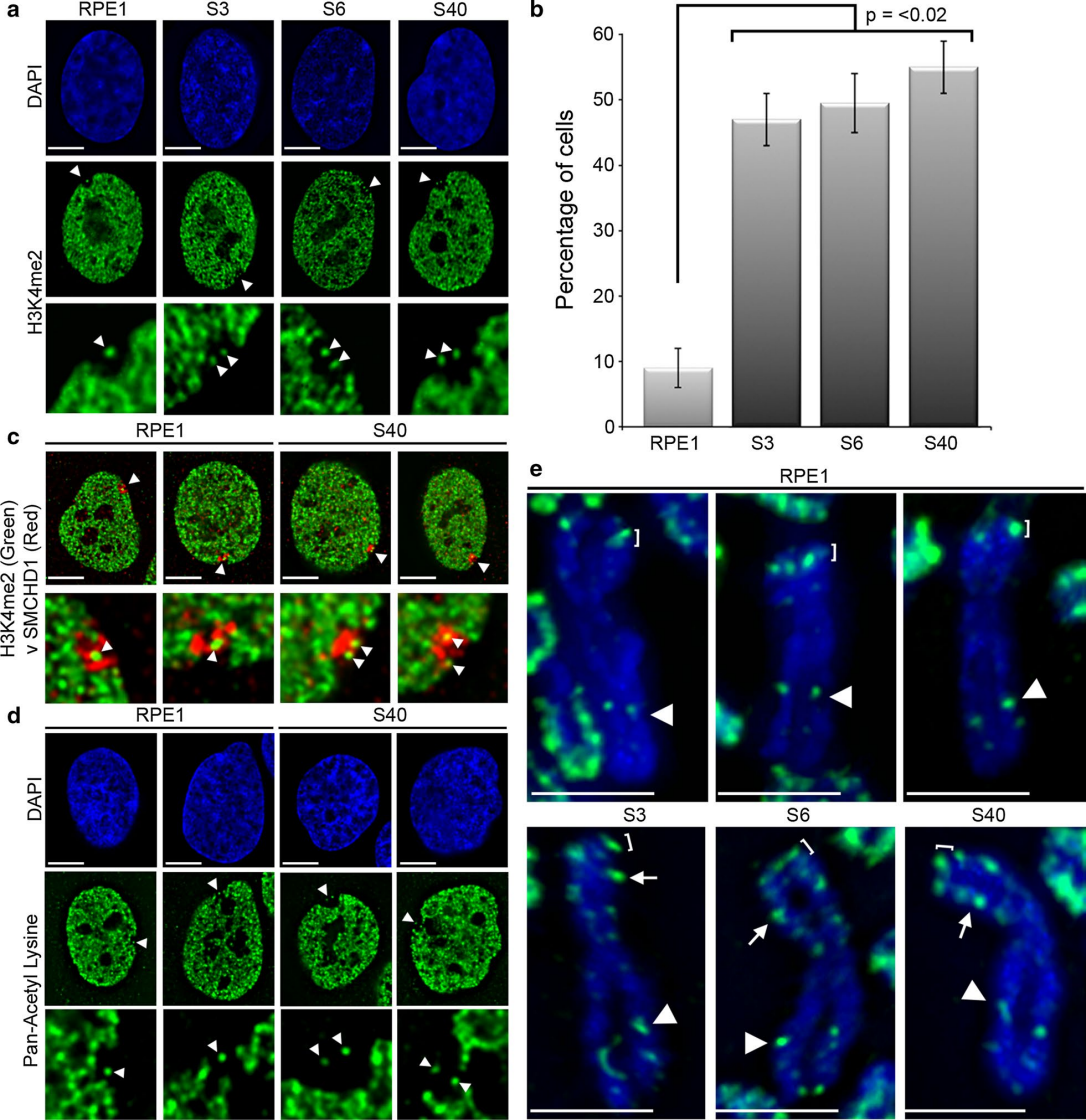
H3K4me2 gain at Xp21 on Xi in SETDB1 mutants. **a** Indirect immunofluorescence showing the distribution of H3K4me2 (Green) in representative RPE1 and SETDB KO nuclei. Nuclei are counterstained with DAPI (Blue). White arrow heads indicate the location of the H3K4me2 dots at the Xi that is expanded under each image. **b** Graph of the percentage of cells with two H3K4me2 foci within the territory of the Xi for RPE1 and three SETDB1 KO clones. Error bars show standard error of the mean. Data represent mean of two replicate data sets, *n* ≥ 150). **c** Indirect immunofluorescence showing H3K4me2 (green) relative to the Xi defined by direct immunofluorescence to SMCHD1 (red). White arrow heads indicate the location of the H3K4me2 dot/s overlapping the Xi SMCHD1 signal that is expanded under each image. **d** Indirect immunofluorescence showing the distribution of pan-acetylated lysine (green) in representative RPE1 and SETDB KO nuclei. Nuclei are counterstained with DAPI (Blue). White arrow heads indicate the location of the Xi-associated acetyl-lysine signal that is expanded under each image. **e** Indirect immunofluorescence showing H3K4me2 distribution on the metaphase Xi in RPE1 and SETDB1 KO clones. White arrow heads point to DXZ4; white brackets indicate the pseudoautosomal associated Xp signal; white arrows indicate the new H3K4me2 Xp band

**Fig. 4:**
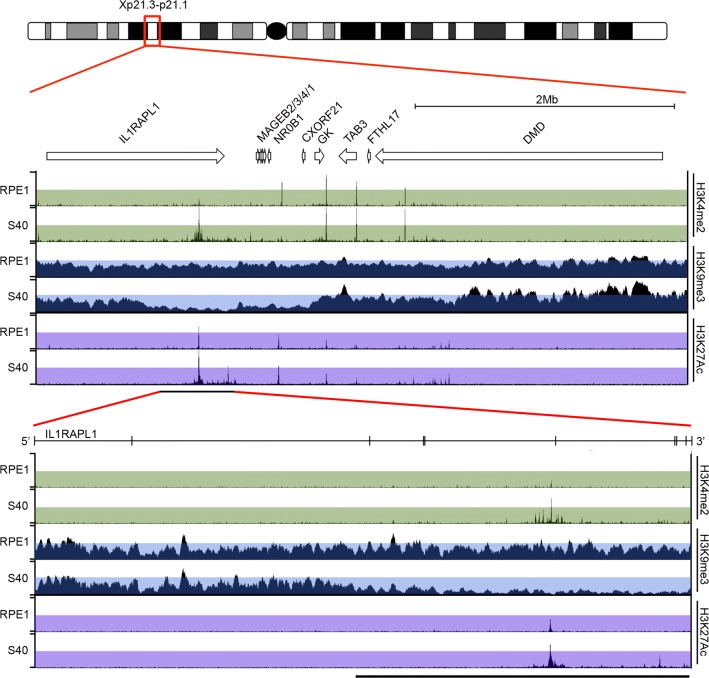
H3K4me2 gain within the 3′ end of the *IL1RAPL1* gene on Xi in SETDB1 mutants. Human X ideogram with Xp21.3-p21.1 boxed and expanded below. White arrows correspond to genes. Beneath is ChIP-Seq profiles for RPE1 and S40 to H3K4me2, H3K9me3 and H3K27Ac (peak heights of 150, 25 and 100, respectively, covering chromosome X: 28,418,150-33,476,294 bp, hg19). The bottom panel is an expansion of the *IL1RAPL1* gene locus (chromosome X: 28,605,681-29,974,017 bp, hg19), showing the same ChIP-Seq profiles (peak heights of 70, 12 and 90 respectively). The *y*-axis on the genome browser image is normalized read density. Colored translucent bands corresponding to the first half of the ChIP-Seq normalized read density have been added to assist in visualizing change regions. The location of *IL1RAPL1* exons is indicated by vertical lines. The black horizontal line at bottom right corresponds to the region of most obvious chromatin profile change. The White horizontal scale bars correspond to 5 μm

Given the decompaction of the Xi in the absence of large-scale facultative heterochromatin change, we next sought to determine whether changes to euchromatin markers at the Xi had occurred. Typically in female nuclei, histone H3 dimethylated at lysine 4 (H3K4me2) shows a general nuclear distribution with obvious underrepresentation at nucleoli and the territory of the Xi that appears as a hole in the staining pattern within which is a single intensely staining focus [2, 23]. This signal was subsequently shown to correspond to the macrosatellite DXZ4 that adopts the opposite chromatin configuration than the flanking chromosome arms: heterochromatin on the Xa and euchromatin on the Xi [24]. While RPE1 cells showed a single focus, in all three mutant clones two intense foci were observed (Fig. 3a) in significantly more cells (Fig. 3b). In addition to DXZ4, nucleosomes of the FIRRE and ICCE tandem repeats are uniquely marked by H3K4me2 at the Xi alleles [25] and, despite residing many megabases apart, these large tandem repeats make frequent Xi-specific contact [25–27]. One possible explanation for the observation of two foci at the Xi could be reduced contact between the three repeat elements accounting the decompaction phenotype. However, none of the tandem repeats overlapped with the new H3K4me2 signal by FISH (data not shown). This suggested that a new euchromatin band had been acquired on the Xi in SETDB1 mutants. To confirm that the H3K4me2 signals reside within the territory of the Xi, we compared the location of the dot/dots with a robust Xi territory marker structural maintenance of chromosomes flexible hinge domain containing 1 (SMCHD1) [28]. Both H3K4me2 dots overlapped SMCHD1 signal at the Xi in the SETDB1-KO clones (Fig. 3c). As a large body of heterochromatin, the Xi is also under represented for histone acetylation [29]. Immunofluorescence to acetylated lysine also clearly defines the territory of the Xi and can detect the euchromatic DXZ4 as a single focus [30]. Like H3K4me2, a single focus of acetylated lysine could be detected within the territory of the Xi in parental RPE1 cells, whereas two foci could be detected in SETDB1-KO cells (Fig. 3d). To identify the origins of this new signal, the distribution of H3K4me2 was determined at metaphase. While RPE1 metaphase Xi showed characteristic intense H3K4me2 bands at DXZ4 and the pseudoautosomal region [23], the Xi in SETDB1-KO clones had an additional intense H3K4me2 band centered at Xp21 (Fig. 3e). Chromatin immunoprecipitation coupled with high-throughput sequencing (ChIP-Seq) was employed in order to fine map the region of chromatin change. Consistent with the metaphase chromosome fluorescence microscopy, a broad interval of approximately 500 kb embedded within the 3′ end of the *IL1RAPL1* gene showed gain of H3K4me2 and a corresponding reduction in H3K9me3 for the same region (Fig. 4). Bacterial artificial chromosomes (BACs) from the chromatin change region were used as FISH probes on metaphase chromosomes stained for H3K4me2. Hybridizing signals overlapped with the new H3K4me2 band at the Xi, whereas the same interval on the Xa appears to lack H3K4me2 signal (Additional file 4). Based on these data, we conclude that loss of SETDB1 results in the acquisition of a broad region of euchromatin within the 3′ end of the *IL1RAPL1* gene on the Xi.

IL1RAPL1 is involved in regulating neuron development [31–35], and mutations in IL1RAPL1 have been reported to cause X-linked intellectual disability [36] and autism spectrum disorder [37], and it is downregulated in brain tumor cell lines and xenografts [38]. Intriguingly, the genomic interval embedded within *IL1RAPL1* that shows chromatin change in the absence of SETDB1 falls within a common fragile site (FRAXC) on the X chromosome [39], indicating that not only is this region susceptible to chromatin transformation but also genomic instability, the two of which may be mechanistically linked.

### IL1RAPL1 transcript levels differ between the 5′ and 3′ ends and are altered in the absence of SETDB1

**Fig. 5:**
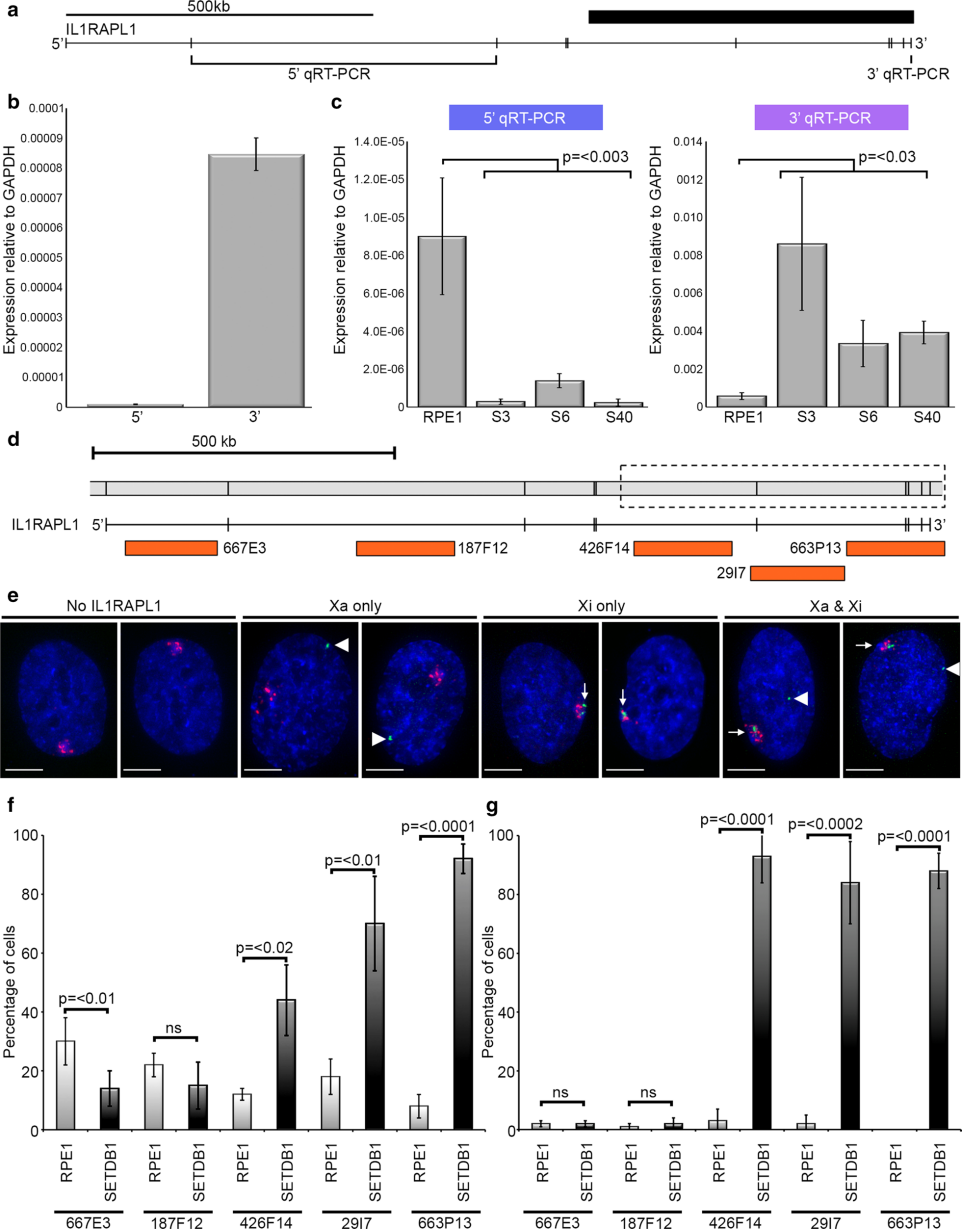
IL1RAPL1 expression changes in SETDB1 KO clones. **a** Map showing the location of *IL1RAPL1* exons (vertical bars) and the regions assessed for the 5′ and 3′ qRT-PCRs. The thick black bar on the right corresponds to the region of chromatin change. **b** Graph showing expression of the 5′ and 3′ ends (x-axis) of the IL1RAPL1 gene relative to GAPDH (*y*-axis) in RPE1 cells as determined by qRT-PCR. Data corresponds to the mean of at least three biological replicates, each performed as three technical replicates. Error bars show standard error of the mean. **c** Graphs showing qRT-PCR results for RPE1 and SETDB1 mutants (*x*-axis) relative to GAPDH expression (*y*-axis). Data for the 5′ qRT-PCR (left) and 3′ qRT-PCR (right) are indicated above. Both qRT-PCR graphs show the mean and standard error for at least three biological replicates, each amplified as three technical replicates. **d** Schematic genomic map of the *IL1RAPL1* locus with the chromatin change interval marked by the dashed box. Orange blocks beneath the gene model correspond to the locations of BAC clones used in FISH experiments. **e** Example images of scored categories of direct-labeled RNA FISH to IL1RAPL1 (green) and XIST (red), merged with DAPI (blue). White arrow heads indicate Xa and white arrows indicate Xi. The white horizontal scale bars correspond to 5 μm. **f** Graph showing the percentage of cells (*y*-axis) with IL1RAPL1 RNA FISH signals at the Xa in RPE1 and SETDB1 KO clones with the five different BAC clones (*x*-axis), and **g** Xi. Scoring data for each clone are given in Additional file 9. All error bars for RNA FISH show standard deviation

In order to determine the impact that the observed euchromatic transition on the Xi may have on expression of IL1RAPL1, we began by further characterizing IL1RAPL1 expression in RPE1 and SETDB1-KO clones. Initially qRT-PCR was performed on cDNA from parental RPE1 cells using oligonucleotide primer sets designed to amplify the 5′ and 3′ end of IL1RAPL1 (Fig. 5a). Unexpectedly, transcript levels at the 3′ end were dramatically higher than the 5′ end (Fig. 5b), suggesting that a substantial proportion of transcripts from the 3′ end may be transcribed from a promoter distinct from the one located at the very 5′ end of the gene. The qRT-PCR analysis was repeated to compare RPE1 and SETDB1-KO clones. Transcripts originating from the 5′ end were significantly reduced in SETDB1-KO clones, whereas transcripts from the 3′ end were significantly elevated (Fig. 5c). To validate these observations and extend them to single-cell analysis, BAC clones from the 5′ and 3′ regions of *IL1RAPL1* were selected for use in RNA FISH experiments (Fig. 5d). RNA FISH was performed with the *IL1RAPL1* BACs in combination with an XIST probe to define the territory of the Xi in the nucleus. Four distinct patterns were observed including no IL1RAPL1 signal, IL1RAPL1 signal distinct from the XIST cloud (interpreted as originating from the Xa), IL1RAPL1 signal associated with the XIST cloud (interpreted as originating from the Xi) and two IL1RAPL1 signals (one associated with the XIST cloud and one distinct, interpreted as being expressed from both alleles) (Fig. 5e). Consistent with the qRT-PCR data, transcripts originating from the 5′ end were significantly reduced in SETDB1-KO clones (Fig. 5f) and almost exclusively originate from the Xa (compare Fig. 5f and g). In contrast, expression from the 3′ end was significantly increased from the Xa (Fig. 5f) and Xi (Fig. 5g) with most transcripts originating from the Xi. Of note, in SETDB1-KO clones 3′ probe RNA FISH detected at the Xi most often appeared as multiple signals interspersed with the XIST cloud, which is in contrast to RNA FISH signals at the Xa that typically were single or double hybridizing foci (most likely due to cells in mid-late S-phase or G2, post-Xa DNA replication) (Fig. 5e). Although at very low incidence, we did occasionally detect 5′ and 3′ RNA FISH signals in proximity to the XIST cloud in parental RPE1 cells, suggesting that under normal circumstances, IL1RAPL1 escapes XCI albeit at substantially lower levels than the expression from the Xa (compare RPE1 between Fig. 5f, g).

### A powerful enhancer located in the 3′ end of *IL1RAPL1* drives bidirectional expression of a novel antisense long noncoding RNA and short sense transcript isoform

These data support a model in which an alternative isoform of IL1RAPL1 originates from the 3′ end of the gene that is dramatically upregulated from the Xa and Xi in the absence of SETDB1. In order to validate this model, we identified four informative single nucleotide polymorphisms (SNPs) within the 3′ transcript [rs140261831, rs113985890, rs190632360 and rs144991617: SNP-A through D, respectively (Additional file 5)]. RPE1 cells are clonal and therefore the chromosomes that were selected as the Xa and Xi are always the Xa and Xi in all cells at all times [40]. Therefore, SNP alleles can be assigned to Xa and Xi, and their presence in RNA indicates their transcriptional origins, although not quantitatively by this approach. Oligonucleotide primers flanking each SNP were designed (Additional file 6) and used to PCR amplify the SNPs in RPE1 DNA and a rodent–human somatic cell hybrid in which the RPE1 Xa is the only human chromosome. Sequencing of the PCR products allowed assignment of SNP alleles to the Xa and Xi. Next, the same intervals were amplified and sequenced from RPE1 and SETDB1-KO clone cDNA. In RPE1 cells, only the Xa allele was detected. This indicates either that Xi 3′ transcript is too low for the SNP allele to be detected by this method or that the observed low-level RNA FISH signals may actually be Xa-derived transcript in the vicinity of the Xi. In contrast, the Xi allele was readily detected in cDNA from SETDB1-KO clones, with minor to moderate contribution of the Xa allele (Additional file 5).

**Fig. 6:**
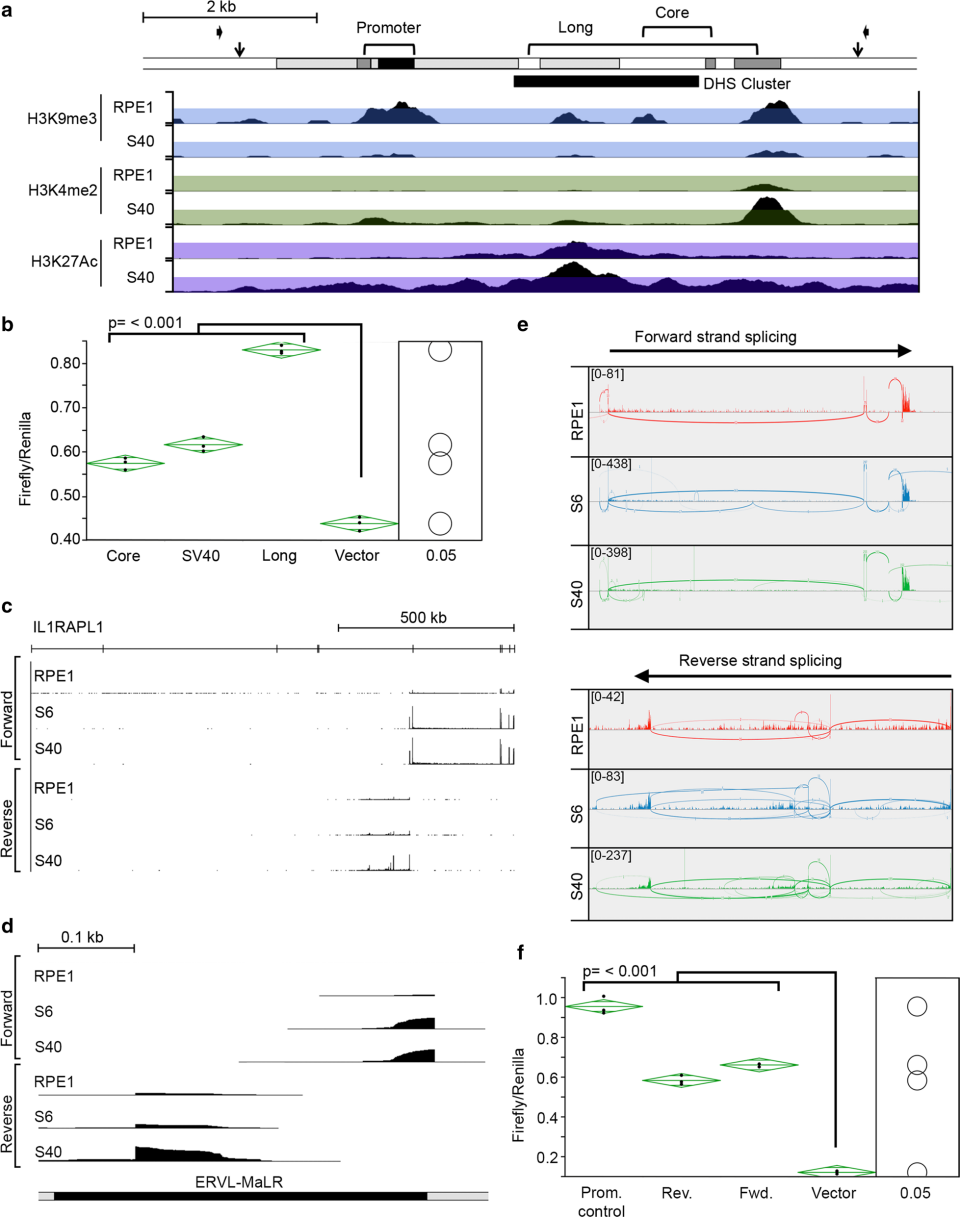
Characterization of the novel enhancer and promoter in the 3′ end of the *IL1RAPL1* gene. **a** Schematic map of the enhancer/promoter region (chromosome X: 29,675,519-29,684,072 bp, hg19). Repetitive elements are shaded. Regions used for the promoter/enhancer (long and core) luciferase assays are indicated. Downward-facing black arrows show the location of gRNAs used for deleting the interval. Inward-facing black arrows correspond to oligonucleotide primers used for PCR screening of mutant clones. Below are ChIP-Seq profiles for RPE1 and S40 to H3K9me3, H3K4me2 and H3K27Ac (peak heights of 10, 150 and 150, respectively). The *y*-axis on the genome browser image is normalized read density. Colored translucent bands corresponding to the first half of the ChIP-Seq normalized read density have been added to assist in visualizing change regions. The location of a DNaseI hypersensitive cluster is indicated. **b** Graph of the results of a dual luciferase assay showing the ratio of Firefly to Renilla activity (*y*-axis) for the indicated enhancer constructs (*x*-axis) displayed as oneway ANOVA. *P* values calculated using Tukey–Kramer honest significant difference. The green diamonds show the mean of the triplicates (central horizontal line) and 95% confidence interval between the apexes of the diamond. Circles on the far right show all pairs Tukey–Kramer 0.05 *p* value spread with significance presented by the angle of intersection between circles. Data shown is representative of data obtained from at least two replicate experiments performed in triplicate. **c** IL1RAPL1 locus RNA-Seq data for RPE1 and SETDB1 KO clones showing forward- and reverse-strand profiles, and **d** profiles centered on the ERVL-MaLR element (black labeled box) (chromosome X: 29,659,850-29,660,300, hg38). **e** Sashimi plots showing quantitative visualization of splice junctions for the novel forward (top, chromosome X: 29,678,310-29,973,937 bp) and reverse (bottom, chromosome X: 29,560,159-29,678,231) transcripts originating from the ERVL-MaLR element for RPE1 and two SETDB KO clones. Boxed numbers in the top left corners indicate exon-coverage data range. **f** Graph of the data for the ERVL-MaLR promoter dual luciferase assay as in part-b above

Next, we set out to identify regulatory elements within the 3′ interval. In addition to H3K4me2 and H3K9me3 ChIP-Seq, we also included histone H3 acetylated at lysine 27 (H3K27Ac) (Fig. 4), a histone modification associated with enhancer activity [41–43]. Numerous novel H3K27Ac peaks were observed spread throughout the 3′ end of *IL1RAPL1* in the SETDB1-KO clones, with the most prominent peak in vicinity to the strongest H3K4me2 peak (Fig. 4). Therefore, we focused our search for regulatory elements on this region. SETDB1-KO clone is associated with H3K4me2 peaks aligned with RPE1 H3K9me3 peaks that were substantially reduced in the mutant clones (Fig. 6a). The major gain of H3K27Ac centered upon a DNaseI hypersensitive site (DHS); regions typically associated with regulatory elements throughout the genome [44]. Within the DNA underlying the H3K27Ac peak was a highly conserved 600 bp sequence. We tested this and a larger sequence underlying the major H3K27Ac peak for enhancer activity in vitro. We observed that the core conserved sequence possessed enhancer activity comparable to the well-characterized and potent SV40 viral enhancer, whereas activity associated with the longer DNA fragment was significantly higher (Fig. 6b), supporting this sequence as a powerful enhancer element.

The enhancer is located downstream of expressed SNPs A and B and upstream of expressed SNPs C and D. Therefore, we sought to determine where this transcript originates through RNA-Seq. To ensure we did not bias toward poly-adenylated mRNA, we resorted to rRNA-depleted strand-specific total RNA-Seq. Through this approach, we identified a sense and antisense transcript originating in the vicinity of the newly identified enhancer element, extending approximately 295 kb in the sense direction and 118 kb antisense, both of which are more prominent in the SETDB1-KO clones (Fig. 6c). Close examination of the most 5′ reads of each transcript revealed that they emanate approximately 80 bp apart and originate within an ERVL-MaLR retrotransposon long terminal repeat (LTR) (Fig. 6d), consistent with promoter activity associated with these elements [45] and their silencing through the activity of SETDB1 [46–48]. The RNA-Seq profiles indicate that both transcripts appear to be spliced, which is confirmed through the generation of Sashimi plots, that allow quantitative visualization of spliced isoforms of transcripts [49]. The forward transcript predominantly incorporates the last 6 exons (exons 6–11) of the full-length *IL1RAPL1* gene, along with a novel first exon (Fig. 6e, top panel). In contrast, all exons associated with the antisense transcript are novel and exon usage is more variable (Fig. 6e, bottom panel). To validate the existence of the novel first exon of the sense transcript and the antisense transcripts, RT-PCR was performed between these exons and downstream exons, generating products of the expected size (Data not shown). The ERVL-MaLR element from within which both first exons originate is located approximately 2 kb proximal to the defined enhancer element (Fig. 6a). To confirm bidirectional promoter activity of the ERVL-MaLR element, it was cloned in both orientations upstream of a promoterless luciferase reporter gene. Activity was slightly higher in the forward orientation when compared to the reverse (Fig. 6f), consistent with the forward transcript being detected at higher levels than the reverse transcript in RPE1 cells (Fig. 6c, e).

### Loss of the ERVL-MaLR promoter and associated enhancer from the Xa decompacts the Xi

These data indicate that loss of SETDB1 results in the derepression of the Xi enhancer and bidirectional promoter embedded within the 3′ end of *IL1RAPL1*, coupled with loss of H3K9me3 and gain of H3K4me2 throughout the extent of the two novel transcripts. Given the dramatic changes that are occurring at this interval, could the reactivation of this powerful enhancer be at least in part responsible for the Xi decompaction phenotype observed in the SETDB1-KO clones? To test this, we used a pair of active guide RNAs (gRNAs) flanking the enhancer and bidirectional promoter with the RNA-guided Cas9 nuclease in an attempt to delete the interval [50]. Numerous clones were isolated in which the desired interval was removed, and assignment of the deletion to the Xa or Xi was achieved by FISH (Additional file 7). PCR coupled with sequencing was performed across each flanking gRNA site on the intact allele to ensure that extensive damage had not occurred in the absence of deletion and in those where this was observed, they were excluded from further analysis (Data not shown).

**Fig. 7:**
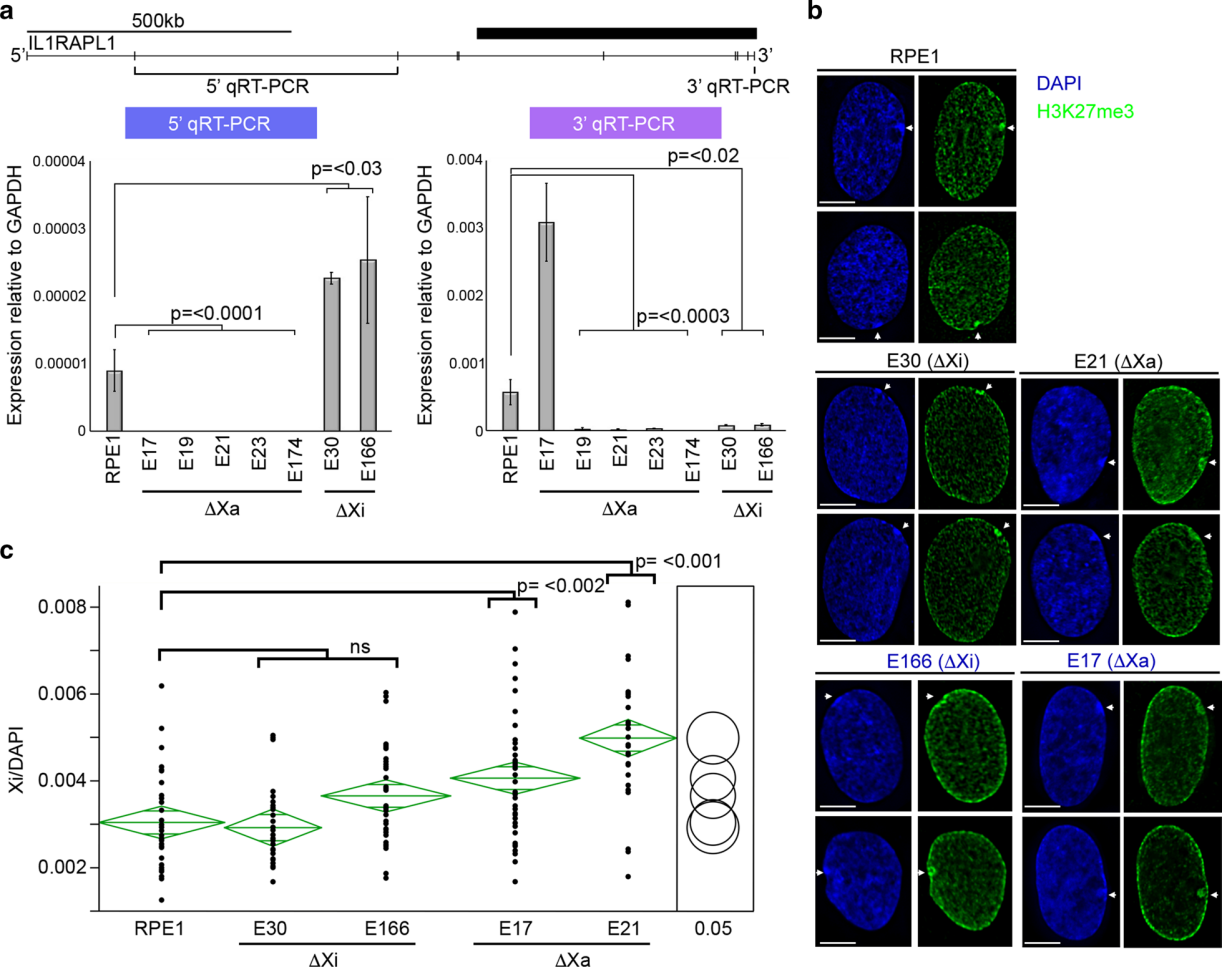
Changes to IL1RAPL1 expression and Xi volume in enhancer deletion mutants. **a** Map showing the location of *IL1RAPL1* exons (vertical bars) and the regions assessed for the 5′ and 3′ qRT-PCRs. The thick black bar on the right corresponds to the region of chromatin change. Immediately beneath the gene model are 5′ (left) and 3′ (right) qRT-PCR results for RPE1 and Xa or Xi enhancer deletion mutants (*x*-axis) showing expression levels relative to GAPDH (*y*-axis). All qRT-PCR graphs show the mean from at least three biological replicates, each performed as three technical replicates. Error bars show standard error of the mean. **b** Representative images of indirect immunofluorescence showing the distribution of H3K27me3 (green) and DAPI (blue) in RPE1 and Xa or Xi IL1RAPL1 enhancer mutants. White arrow heads indicate the location of the Xi. The white horizontal bars correspond to 5 μm. **c** Volume measurements of the Xi in RPE1 compared to Xa or Xi IL1RAPL1 enhancer deletion mutants displayed as oneway ANOVA. *P* values calculated using Tukey–Kramer honest significant difference. Xi/DAPI volumes are indicated on the *y*-axis. Each black dot indicates a measurement made for individual nuclei in each sample. The green diamonds show the mean (central horizontal line) and 95% confidence interval between the apexes of the diamond. The width of the diamond is proportional to the sample size with wider diamonds indicating more measured nuclei. Circles on the far right show all pairs Tukey–Kramer 0.05 *p* value spread with significance presented by the angle of intersection between circles

To further characterize these mutants, we initially assessed expression levels of IL1RAPL1 from the 5′ and 3′ ends by qRT-PCR. Interestingly, loss of the enhancer element from the Xa resulted in compete loss of 5′ transcript that initiates transcription from a promoter located greater than 1 Mb upstream. This indicates that transcription from the 5′ promoter is dependent upon the enhancer element. Interestingly, loss of the enhancer element from the Xi resulted in an increase in expression from the 5′ end that surprisingly corresponds to upregulation from the Xa in trans (see below). With the exception of one Xa enhancer mutant, expression from the 3′ end decreased, regardless of whether the enhancer was deleted from the Xa or Xi (Fig. 7a). In order to determine the predominant origins of the 3′ transcripts, cDNA isolated from the mutants was genotyped for two of the SNPs. As expected, only the Xa SNP could be detected in 3′ transcripts from Xi deletion mutants. However, surprisingly the 3′ transcripts detected in Xa enhancer mutants only showed the Xi allele, suggesting that, like the SETDB1-KO clones, the Xi enhancer and bidirectional promoter were active in these clones (Additional file 7). If the Xi enhancer was indeed activated in these clones, we might expect to see some degree of decompaction of the Xi, mimicking the SETDB1-KO phenotype. Staining of enhancer mutant cells for H3K27me3 showed that the Xi territory was not obviously different in the Xi enhancer mutants but was noticeably larger in clones lacking the enhancer from the Xa (Fig. 7b). Subsequent volume measurements of the Xi in these clones confirmed that loss of the enhancer from the Xa, but not Xi, resulted in a significant decompaction of the Xi territory (Fig. 7c). Collectively, these data reveal complex cis and trans effects upon deletion of the enhancer interval.

## Discussion

SETDB1 loss in human cells does not result in chromosome-wide changes to H3K9me3 bands along the Xi, although a role in establishing these territories during XCI similar to what has been described in mouse cannot be ruled out [7]. However, testing XCI onset questions in human cells in vitro faces many technical challenges [51]. Which HMTase is responsible for maintenance of H3K9me3 at the human Xi remains an open question, although functional redundancy between this group of enzymes remains a possibility. Nevertheless, we find that SETDB1 loss does result in a striking Xi decompaction phenotype based on H3K27me3 volume measurements. The overall distribution of H3K27me3 at the Xi did not change, ruling out spread of this modification beyond its normal sites of occupancy, and therefore decompaction may reflect changes to the interchromatin compartment organization at the Xi [52]. To our knowledge, the only other report of Xi decompaction involves knockdown of the heterochromatin proteins SMCHD1 and LRIF1 [53]. Whether SETDB1 is acting in the same pathway as these proteins has yet to be determined.

Alongside the decompaction phenotype, we observed that loss of SETDB1 triggered a large-scale chromatin state transition on the Xi within an approximately 500 kb interval located in the 3′ end of the *IL1RAPL1* gene. H3K9me3 was lost coupled with gain of H3K4me2 and broad acquisition of H3K27Ac throughout the interval. The highest peaks of H3K27Ac and H3K4me2 reside near the center of the chromatin change interval, and the DNA underlying these peaks correspond to a powerful enhancer adjacent to an ERVL-MaLR element that promotes bidirectional transcription. While transcription in the reverse direction produces a novel long noncoding RNA (lncRNA), the forward transcript has the potential to generate a protein isoform of IL1RAPL1 that lacks part of the N-terminus. Both transcripts are abundantly transcribed from the Xi in SETDB1-KO clones and appear interspersed with the XIST cloud. Given the central role of SETDB1 in silencing endogenous retroviral elements [46–48], we interpret these data to indicate that under normal circumstances, SETDB1 is recruited to the ERVL-MaLR element at the Xi and facilitates heterochromatin formation and maintenance, but in its absence H3K9me3 is lost permitting reactivation of the LTR and adjacent enhancer driving escape from XCI. In contrast, the Xa allele is unlikely to be repressed by SETDB1 as the enhancer is essential to permit transcription of IL1RAPL1 from the promoter located at the 5′ end of the gene.

Interestingly, mutations in or loss of SETDB1 have been reported in cancer [54, 55]. Based on our data, we would predict that full-length IL1RAPL1 would be downregulated, which is indeed the case in brain tumors [38]. Notably, no other regions of the Xi showed such chromatin instability, while only speculating, it has not escaped our notice that this same interval is embedded within FRAXC, a common fragile site on the X [38], indicating that it is also a region of genomic instability. Mutation or loss of *IL1RAPL1* results in intellectual disability [36] and the unstable chromatin transition interval is almost always lost or rearranged in patients [36, 56–61], suggesting that the two may be mechanistically linked. The DNA sequence of FRAXC does not hold clues as to why this region is fragile, but perhaps its chromatin state might. Furthermore, IL1RAPL1 genomic instability has been implicated in numerous other neurological and neuropsychiatric disorders including startle epilepsy [62], autism spectrum disorder [37, 63] and schizophrenia [64]. The link between SETDB1 and neurological disorders extends beyond what is described here for IL1RAPL1, as neuronal ablation of SETDB1/Setdb1 results in heterochromatin loss and disrupted expression at the protocadherin cluster [65].

**Fig. 8:**
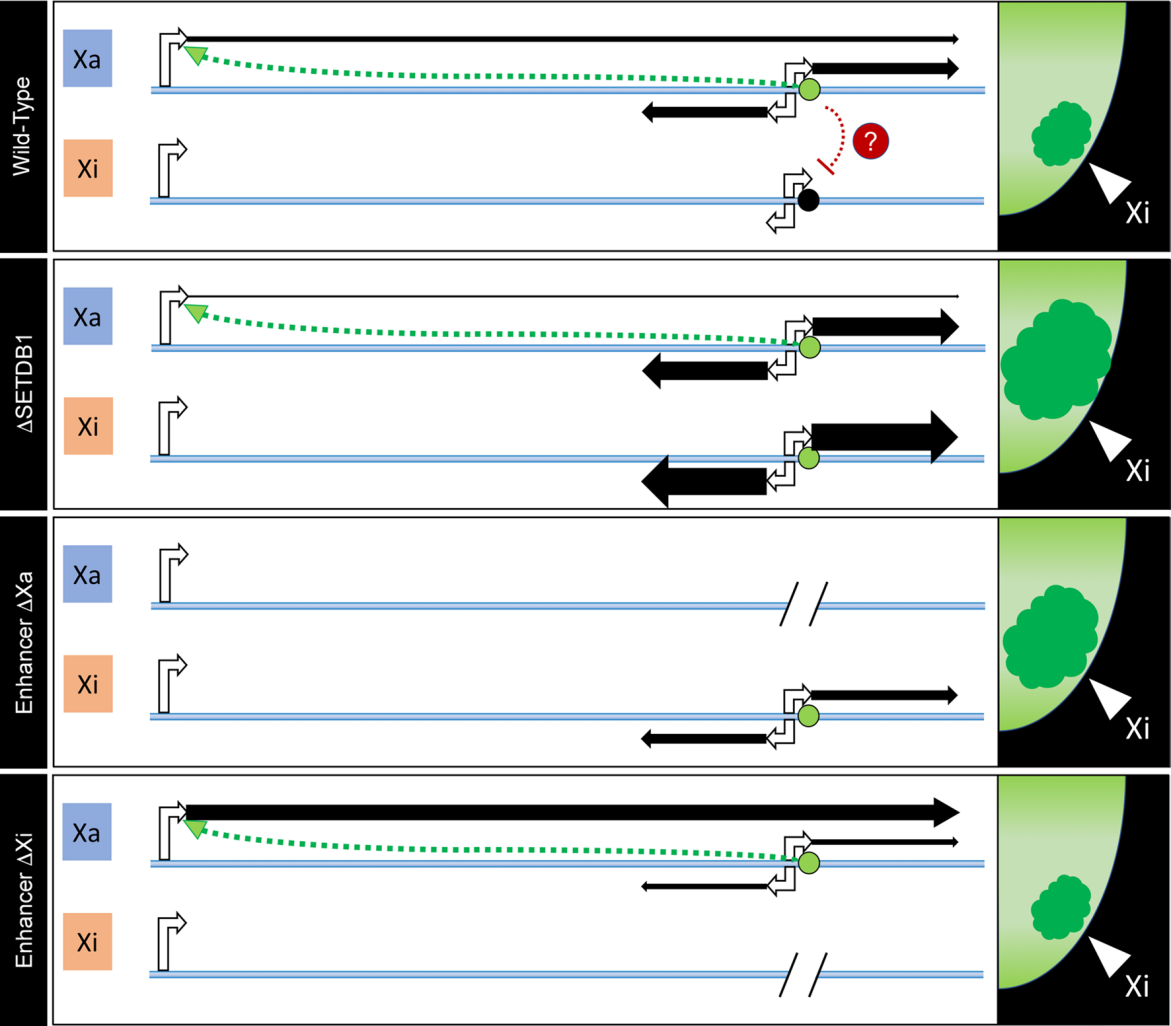
Model summarizing observations made at the Xi and *IL1RAPL1* locus in parental and mutant clones. In each of the four panels, the blue horizontal bar represents the Xa and Xi alleles of the *IL1RAPL1* gene locus in the parental or mutant clones as indicated to the left. The hooked white arrow at the far left represents the main promoter, whereas the two divergent arrows to the right represent the bidirectional ERVL-MaLR promoter that is immediately adjacent to the enhancer element (circle; green = active, black = silent). Black arrows represent transcript and the relative thickness represents the amount of transcript. The green dashed arrow represents the cis promoter activation from the enhancer. The red dashed line and question mark represent the potential for trans effects. To the right of each panel is a zoomed in cartoon schematic of a female nucleus showing H3K27me3 signal in green with the dark green region representing the territory of the Xi

The reactivation of the powerful enhancer coupled with adjacent bidirectional expression on the Xi upon SETDB1 loss raised the possibility that this could be mechanistically linked to the Xi decompaction phenotype. Deletion of the enhancer from the Xi in the absence of SETDB1 mutation did not result in Xi decompaction, but deletion of the enhancer from the Xa did, which was coupled with detection of 3′ bidirectional transcripts from the Xi only. How this trans-chromosomal effect is mediated is unclear but was not a phenomenon restricted to the Xa deletion impacting the Xi, because cells with the enhancer deleted from the Xi showed increased expression on the Xa from the 5′ end of the gene. A summary of our findings is outlined in Fig. 8. In parental cells (top panel), IL1RAPL1 expression is almost exclusively originating from the Xa, both from the 5′ promoter and the bidirectional ERVL-MaLR promoter. The adjacent enhancer element promotes expression of the 5′ transcript in cis. Upon loss of SETDB1 (second panel), local heterochromatin around the enhancer and adjacent bidirectional promoter are lost at the Xi resulting in activation of the enhancer and 3′ transcription. Heterochromatin loss does not extend sufficiently far to permit activation of the Xi 5′ promoter. The enhancer and ERVL-MaLR reactivation is coupled with dramatic decompaction of the Xi territory by unknown mechanisms. Deletion of the bidirectional promoter and enhancer from the Xa (third panel) results in loss of cis-mediated activation of the 5′ promoter coupled with inappropriate activation of the Xi bidirectional promoter as well as substantial decompaction of the Xi territory. In contrast, loss of the enhancer and bidirectional promoter from the Xi (bottom panel) does not impact Xi compaction, but does result in a significant increase in expression from the 5′ promoter on the Xa coupled with reduced 3′ transcription. How these trans-allele effects are mediated is unclear. An attractive candidate are the transcripts generated from the 3′ end, especially the antisense lncRNA, as trans-induction effects of enhancer-associated lncRNA have been reported [66, 67]. One possibility may be posttranscriptional regulation of the 5′ transcript, as the levels of antisense lncRNA appear to be indirectly correlated with 5′ derived transcript levels. To decipher exactly what the underlying mechanisms are is deserving of further investigation as is exploring this novel enhancer element in the context of neuronal function. While we recognize that the decompaction of the Xi in the Xa enhancer deletion mutants is not as extensive as observed in the SETDB1-KO clones, it is a major contributor to the decompaction phenotype. What other factors are involved as well as how and why the Xi decompacts remain to be determined.

## Conclusions

Collectively we reveal a novel critical function for SETDB1 in ensuring the proper compaction of the human Xi chromosome territory through the silencing and seeding of heterochromatin at an ERVL-MaLR LTR and adjacent enhancer in the 3′ end of the *IL1RAPL1* locus on the Xi allele. Through targeted deletion of the ERVL-MaLR element and enhancer, we reveal unexpected cis and trans effects impacting chromosome compaction and transcription of IL1RAPL1 isoforms. In light of these new data, further investigation of these findings in the context of genomic instability, neurological disorders and expression changes in brain tumors is warranted.

## Methods

### Cell culture

hTERT-RPE1 (RPE1), a female telomerase-immortalized human retinal pigment epithelial cell line, was obtained from Clontech (C4000-1). This product is no longer available through Clontech but can be obtained directly through the American Type Culture Collection (ATCC). Human female embryonic kidney cell line 293 (CRL-1573) was obtained from ATCC. Cells were maintained according to the supplier recommendations. Transfection of plasmid DNA into 293 cells was performed using Lipofectamine 3000 (Cat. No. L3000008) according to the supplier instructions (ThermoFisher Scientific). TALEN expression plasmids and targeting constructs were introduced into RPE1 cells using a 4D-Nucleofector (Lonza) using SF Cell Line 4D-Nucleofector reagent (Cat. No. V4XC-2024), as were gRNA/Cas9 expression constructs.

### TALEN and gRNA/Cas9 targeting

TALEN pairs previously reported to actively cut the human SETDB1 locus were assembled using the FLASH procedure essentially as described [21]. Activity of the TALENs was validated using the Surveyor assay (Transgenomic) [68]. The homology-mediated repair promoterless neomycin trap construct was generated by four-way cloning of a left and right homology arm fragment with the pSEPT vector into pAAV [22, 69]. The left homology arm was generated by PCR of a 1217 bp genomic fragment proximal to SETDB1 exon-2 incorporating a NotI site at the most distal edge and a XbaI site at the proximal edge. The right homology arm was generated by PCR of a 785 bp genomic fragment distal to SETDB1 exon-2 incorporating a EcoRI site at the most proximal edge and a NotI site at the distal edge. Restriction enzyme sites were introduced into the PCR products by including the recognition sequences at the 5′ end of the oligonucleotide design. Homology arm PCR fragments were TA cloned into pCR2.1 (ThermoFisher Scientific), and clones lacking mutations were selected. Homology arm inserts were excised with XbaI-NotI or EcoRI-NotI, respectively, and gel purified along with gel purification of the XbaI-EcoRI pSEPT neomycin fragment. These fragments were mixed with NotI-cut phosphatase-treated pAAV and ligated for 1 h at room temperature before transformation into competent DH10B before plating on ampicillin-containing LB agar plates. Transfection-grade plasmid DNA was isolated from a positive clone and linearized with SmaI before introduction alongside TALEN constructs into 1 million RPE1 cells by Nucleofection. Twenty-four hours later, the cells were manually seeded to four 96-well plates and allowed to attach overnight before supplementing the media with neomycin (580 ng/μl). Three to four weeks later, colonies were expanded and genomic DNA isolated. The DNA was used in two initial rounds of PCR (Additional files 1 and 2). The first PCR used a forward primer located proximal to the left homology arm and a reverse primer located in pSEPT. The second PCR used a forward primer located in pSEPT and a reverse primer located distal to the right homology arm. Successfully targeted clones were positive to both of these PCRs and were subjected to a third PCR. The third PCR amplified a 269 bp fragment spanning the TALEN cut sites. Samples negative for this PCR (but positive for an unrelated PCR of similar size) are interpreted as successfully targeted at both alleles. Those clones positive for this PCR were repeated and the product sequenced to determine whether any mutations from non-homologous end joining had been introduced at the cut site.

Sequences flanking the putative IL1RAPL1 enhancer core were used to identify suitable gRNAs using the Optimized CRISPR Design (http://crispr.mit.edu) and cloned into pSpCas9(BB)-2A-GFP (pX458) as described [50]. Paired combinations of gRNA constructs capable of excising the intervening sequence were identified by co-transfection experiments into 293 cells followed by isolation of genomic DNA using the NucleoSpin Tissue kit from Macherey–Nagel (Cat. No. 740952). Genomic DNA was used to perform PCR across the interval to determine which combination of gRNAs was most efficient at generating the desired deletion. Transfection-grade plasmid DNA was isolated for the desired pair of gRNA expression constructs (Qiagen, Cat. No. 12945) and was introduced into RPE1 cells by Nucleofection. Seventy-two hours later, single GFP positive cells were directly sorted into single wells of four 96-well plates using a BD FACSAria SORP flow cytometer. Colonies were expanded 3–4 weeks later and assessed for the presence or absence of the desired deletion by PCR. Assignment of the deletion to the Xa or Xi was performed by hybridizing a probe contained within the deleted region onto dropped metaphase chromosomes (Additional file 7). The Xa can be cytologically distinguished from the Xi due to an X:10 translocation at Xq28 on the Xa making this chromosome substantially longer than the Xi. The karyotypically normal X is the Xi in all RPE1 cells [30].

### Immunofluorescence on interphase cells and metaphase chromosomes

Indirect immunofluorescence on interphase cells was performed essentially as described previously [70] and on metaphase chromosomes [6]. Direct labeling of primary antibodies was achieved using the Zenon Antibody Labeling Kit from ThermoFisher Scientific according to the supplied instructions (Cat. No. Z2305).

### DNA and RNA FISH on interphase cells and metaphase chromosomes

Dropped metaphase chromosome spreads and subsequent DNA FISH were performed essentially as described [70]. Direct-labeled fluorescent probes were prepared using the Nick Translation DNA Labeling System from Enzo Lifesciences (Cat. No. ENZ-42910) with fluorescent deoxynucleotides Spectrum Orange and Spectrum Green (Abbott Molecular, Cat. Nos. 02N32-050 and 02N33-050). RNA FISH was performed as described previously [71]. The identity of BAC clones used for FISH is given in Additional file 8.

### Antibodies

Rabbit polyclonal antibodies to histone H3 trimethylated at lysine 27 were obtained from EMD Millipore (Cat. No. 07-449), as were rabbit polyclonal antibodies to histone H3 trimethylated at lysine 9 (Cat. No. 07-523), histone H3 dimethylated at lysine 4 (07-0030), anti-acetyl-lysine (06-933) and mouse monoclonal antibodies to HP1β (Cat. No. MAB3448). Alexa-Fluor-conjugated secondary antibodies were obtained from ThermoFisher Scientific. Rabbit polyclonal antibodies to SMCHD1 were obtained from Bethyl Laboratories Inc. (Cat. No. A302-872A). Rabbit antibodies to β-actin were obtained from Cell Signaling Technology (Cat. No. 4970). Rabbit polyclonal antibodies to SETDB1 were obtained from Cell Applications Inc. (Cat. No. CY1112).

### Western blotting

Protein extracts, polyacrylamide gel electrophoresis, Western blotting and detection were carried out as described previously [72].

### Quantitative RT-PCR analysis and standard PCR

All qRT-PCR was performed on a CFX96 (Bio-Rad) using an EvaGreen 2X qPCR mastermix (ABM, Cat. No. Mastermix-S) as described previously [72]. Standard endpoint PCR analysis used HotStar Taq Plus (Qiagen) according to the supplier recommendation (Cat. No. 203603). All oligonucleotides were obtained from Eurofins Genomics and are given in Additional file 6.

### ChIP-Seq

ChIP-Seq to H3K4me2, H3K27Ac and H3K9me3 was performed through the full-service procedure provided by Zymo Research according to their established protocols. This included the ChIP assay, library preparation, Next-Gen 50 bp single-end sequencing, bioinformatics and all quality control steps. Three independent frozen pellets of 5 million formaldehyde cross-linked cells for each sample were provided to Zymo Research for the ChIP procedure. In brief, cells were cross-linked with formaldehyde at 1% final concentration for 7 min at room temperature. ChIP assays were conducted using the Zymo-Spin ChIP kit (Zymo Research Corp., Cat. No. D5210) following the manufacturer’s instructions. Sonication was performed at high power setting for 40 cycles (30 s on, 30 s off) using a Bioruptor Plus (Diagenode Inc., Denville, NJ), yielding a modal fragment size of < 600 bp. Antibodies used in ChIP assays included: anti-H3K4me2 (Abcam ab7766), anti-H3K9me3 (Abcam ab8898), anti-H3K27ac (Abcam ab4729) and normal rabbit IgG (Millipore 12-370 or Diagenode C15410206). Approximately 10 µg of chromatin from RPE1 or SETDB1 cells was used in each ChIP assay with 4 µg of antibodies. IgG negative control was included with each assay. DNA libraries were prepared by Zymo Research Epigenetics Services and were sequenced on a HiSeq sequencer. ChIP-Seq reads were aligned to human reference genome GRCh37/hg19 by Bowtie [73] with at most two mismatches. Reads that appeared more than twice at the same position on the same strand were discarded to remove PCR duplication. WIG files were generated from the coverage for visualization purposes. MACS2 [74] was used to identify target peaks at a q-value cutoff 0.01 using narrow peak for H3K4me2 and H3K27ac and broad peak setting for H3K9me3 using normalized mapped sequencing reads. In addition, target peaks overlapping by more than 50% were considered “common” to both samples; otherwise, both of these two peaks would be considered “unique.”

### RNA-Seq

RNA-Seq was performed through the services provided by Novogene. Triplicate total RNA extracts for each clone were prepared using the Nucleospin RNA Plus kit from Macherey–Nagel (Cat. No. 740984). For each sample, 6 μg of total RNA was provided to Novogene who then performed quality control, removed rRNA and prepared strand-specific libraries before generating at least 60 million 150 bp paired-end reads per sample. Adapter sequences were removed using Cutadapt [75]; Fastq stranded data files were mapped to the plus or minus strand of human genome build hg38 using HISAT2 [76]. SAM files were converted to BAM files using SAMtools [77] and BAM files were converted to bedGraph files using BEDTools [78] before conversion to bigWig format for viewing on the UCSC Genome Browser (https://genome.ucsc.edu/index.html). Sashimi plots were generated using the function provided by the Integrative Genomics Viewer (IGV_2.3.97).

### Sanger sequencing

All Sanger sequencing was performed using the services provided by Eurofins Genomics, and sequences were edited and visualized using Sequencher 5.4.6 (Gene Codes Corporation).

### Luciferase promoter and enhancer assays

A DNA fragment of 810 bp encompassing the core conserved putative enhancer region of IL1RAPL1 and a larger fragment of 2613 bp were amplified with primers that incorporate a 5′ SalI and 3′ BamHI site before cloning into pMiniT2.0 (NEB, Cat. No. E1202S). Clones were verified to be free of PCR-induced mutations before subcloning into the SalI-BamHI sites of pGL4.23 (Promega, Cat. No. E8411) downstream of the luciferase expression cassette. As an enhancer control, the SV40 enhancer fragment of pGL3.control (Promega, Cat. No. E1741) was excised with XbaI and BamHI and subcloned into XbaI-BamHI cut pGl4.23. A DNA fragment of 529 bp encompassing the ERVL putative promoter element was amplified and cloned into pMiniT2.0. Clones were verified to be free of mutations by sequencing, and the insert was excised using XhoI and subcloned into XhoI cut and de-phosphorylated pGL4.10 (Promega, Cat. No. E6651). Clones were isolated that contained the ERVL element in either orientation. Dual luciferase assays were carried out as described previously [25]. Empty vectors served as negative controls, whereas the SV40 enhancer construct served as a positive control for the enhancer assay and a previously characterized promoter construct for the DANT2 lncRNA [79] served as a promoter positive control.

### Bacterial artificial chromosomes

All BAC clones were obtained from ThermoFisher Scientific. Clones were cultured according to the supplier recommendations and DNA isolated using the NucleoBond Xtra BAC isolation kit from Macherey–Nagel (Cat. No. 740436). BAC clone information is given in Additional file 8.

### Image acquisition and volume measurements

Images were collected on a Delta Vision pDV. Images were de-convolved with softWoRx 6.5.2 (GE Healthcare) and compiled into images using Adobe Photoshop CC 2018 (Adobe Systems). For nuclear volume, de-convolved D3D files were used with Model-2D polygon finder, selecting a rectangle box around the DAPI image with a minimum perimeter setting of 300. 3D object builder was used to generate 3D objects followed by the Measurements function to generate a table of 3D measurements. H3K27me3 Xi volumes were measured as above setting the minimum perimeter to 30. The Xi to DAPI ratio was calculated in Microsoft Excel before importing into JMP pro 12 for statistical analysis of volume measurement. Typically, 40 nuclei are assessed for each sample.

### Statistics analysis

Unpaired *t* tests were performed using the QuickCalcs function in GraphPad software. JMP Pro 12 was used for the statistical analysis of Xi volume measurements and luciferase assay data. Means of all pairs were compared using the Tukey’s honest significance test.

## Additional files

**Additional file 1.** TALEN-assisted targeting of the SETDB1 gene. (**a**) Inverted ethidium bromide-stained agarose gel image showing the results of the Surveyor assay. 293 cells were transiently transfected with mammalian expression constructs expressing a previously reported active TALEN pair targeted to exon-2 of human SETDB1 [21] (SETDB1), the same TALEN pair assembled using an alternative platform [80] (SETDB1-Alt) and a transfection without DNA (Mock). Two days post-transfection, DNA was isolated from the cells and PCR performed across the cut site before performing the Surveyor assay. The right-facing black arrow head indicates the successful generation of Indels at exon-2 of SETDB1 in the SETDB1-TALEN transfection but not the alternate platform (SETDB1-Alt) or negative control mock sample. (**b**) Schematic representation showing the relative position of exons 1–3 of *SETDB1*. Gray-shaded intervals represent repetitive DNA sequences, whereas white regions are unique. The location of the left and right homology arms (LHA and RHA) are shown in pink above the genomic locus. Upward- and downward-facing black arrow heads indicate the binding location of the top-strand binding and bottom-strand binding TALENs. Inward-facing black arrows represent the location of oligonucleotide primers used for the Surveyor PCR. The location of exons 1–3 is shown as black boxes (exons) joined by black lines (introns) below the map. (**c**) Schematic representation of a successfully promoter trap targeted *SETDB1* locus. The blue shaded interval represents the integrated pSEPT vector cloned between the LHA and RHA that contains the promoterless neomycin cassette. Inward-facing arrows show the approximate location of oligonucleotide primers used to amplify between the pSEPT vector and genomic locations up and downstream of the homology arms that are used for screening of clones by PCR. Beneath the map is a schematic showing the splicing of exon-1 to the neomycin cassette followed by transcription termination by a polyadenylation signal located in the pSEPT vector.

**Additional file 2.** PCR screening for SETDB1-targeted clones. Representative examples of PCR screening results in searching for *SETDB1* targeting. Each image shows an ethidium bromide-stained agarose gel with results for correct left homology arm integration (top), right homology arm integration (bottom) and the presence or absence of the TALEN cut site (middle). Molecular weight markers are in the first lane of each gel, and the sizes of fragments in kb are indicated to the left. PCR results for independent clones are shown in each lane and labeled above. Negative controls of RPE1 genomic DNA and water are shown at the far right of the gels. Successfully targeted clones generate a 1512 bp product for the left side and a 2183 bp product for the right side. The TALEN cut-site PCR generates a 269 bp product if the interval is intact or only targeted at one *SETDB1* allele. Clones 3, 6 and 40 were selected for further analysis. All three are positive for the correct left and right targeting and are negative for the cut site PCR.

**Additional file 3.** Nuclear volume measurements. Graph shows the results of nuclear volume measurements (based on DAPI volume) in RPE1 alongside the three SETDB1 mutants, S3, S6 and S40 (*x*-axis). DAPI volumes are indicated on the *y*-axis. Each black dot indicates a measurement made for individual nuclei in each sample. The green diamonds show the mean (central horizontal line) and 95% confidence interval between the apexes of the diamond. The width of the diamond is proportional to the sample size with wider diamonds indicating more measured nuclei. Circles on the far right show all pairs Tukey–Kramer 0.05 *p* value spread with significance presented by the angle of intersection between circles. None of the SETDB1 mutants have nuclear volumes that are significantly bigger than parental RPE1.

**Additional file 4.** Validation of the SETDB1 mutant-specific H3K4me2 Xi band within the 3′ end of the *IL1RAPL1* gene. (**a**) Schematic map of the *IL1RAPL1* gene locus. The horizontal line represents introns bisected by vertical lines corresponding to exons. The solid black bar beneath the map indicates the approximate extent of chromatin change observed by ChIP-Seq (Fig. 2d). The orange bars represent the location of the indicated BAC clones. (**b**) Top panel shows representative examples of metaphase Xi in SETDB1 mutant S40 showing the distribution of H3K4me2 by indirect immunofluorescence (red) merged with DAPI (Blue). White arrow heads indicate the location of the novel H3K4me2 band observed in the mutant clones. The second row shows the hybridizing BAC probe signal (green) merged with DAPI (blue), whereas the last row shows a merge of all three. (**c**) Top panel shows an example of the H3K4me2 indirect immunofluorescence pattern (red) merged with DAPI (Blue) on the Xa alongside the Xi. The Xa-specific translocation of chromosome 10 at Xq28 results in a substantially longer Xa relative to the Xi, facilitating the ability to readily distinguish the two chromosomes. White arrow heads indicate the presence of the H3K4me2 band on the Xi but not Xa. The second panel shows the hybridization pattern for BAC probe 426F14 (Green) merged with DAPI (Blue). The bottom panels show a merge of all three.

**Additional file 5.** Genotyping results for transcripts originating from the 3′ end of *IL1RAPL1*. (**a**) Schematic map of the 3′ end of IL1RAPL1. The gray bar represents the genomic locus and black vertical lines indicate exons. The relative location of SNPs A–D is indicated above the genomic map. Below the genomic map is a representation of the IL1RAPL1 primary transcript followed by the relative position of BAC clones. (**b**) Inverted ethidium bromide-stained agarose gel image showing RT-PCR results with primers spanning SNPs A, B, C and D. Each gel shows RT-PCR results for a negative control water sample, cDNA prepared with reverse transcriptase (+RT) and without reverse transcriptase (− RT) for parental RPE1 and SETDB1 mutant S40. (**c**) DNA sequence traces for SNPs A, B, C and D in RPE1 genomic DNA, genomic DNA isolated from a somatic cell hybrid in which the RPE1 Xa is the only human chromosome present, and cDNA samples from RPE1 and SETDB1 mutant S40. The downward-facing black arrow heads at the top indicate the location of the SNP that appears as two merged traces in samples containing material from both alleles.

**Additional file 6.** List of oligonucleotides used in this study. All oligos were obtained from Eurofins Genomics LLC. Oligo names are given in column-1, the corresponding sequence is in column-2, and what the oligo was used for is listed in column-3.

**Additional file 7.** Validation of successful deletion of the IL1RAPL1 enhancer and the impact on allele expression by SNP genotyping. (**a**) Ethidium bromide-stained agarose gel images showing PCR results from individual clones exposed transiently to the gRNA pair flanking the enhancer locus. Successful deletion of the enhancer interval is indicated by the presence of a PCR product. Parental RPE1 and water are negative controls, whereas the “Pool Sorted” sample represents a pool of FACS sorted RPE1 cells exposed to the gRNAs as a positive control. (**b**) Dropped metaphase chromosome spreads for the indicated clones showing the hybridizing FISH signals for Xp control BAC probe ZFX (Red) and a probe generated from the enhancer deleted region (Green) merged with DAPI (Blue). The Xa and Xi are indicated. (**c**) DNA sequence traces for SNPs A and C in RPE1 genomic DNA, genomic DNA isolated from a somatic cell hybrid in which the RPE1 Xa is the only human chromosome present, and cDNA samples from RPE1, SETDB1 mutant S40 and four independent enhancer mutants. The downward-facing black arrow heads at the top indicate the location of the SNP that appears as two merged traces in samples containing material from both alleles.

**Additional file 8.** List of BAC clones used in this study. BAC clone names are given in column-1 and the corresponding gene content is listed in column-2

**Additional file 9.** IL1RAPL1 RNA FISH data for the BACs indicated in the cells listed. Xi expression is defined as a single associated with the XIST RNA cloud. Each column represents data from replicate experiments. Therefore, a clone with BAC data in two columns indicates that the experiment was done twice, whereas that in four columns indicates the experiment was done on four occasions

## Abbreviations

BAC: bacterial artificial chromosomes
ChIP-Seq: chromatin immunoprecipitation coupled with high-throughput sequencing
DHS: DNase I hypersensitive site
EZH2: enhancer of Zeste 2
FISH: fluorescent in situ hybridization
FLASH: fast ligation-based automatable solid-phase high-throughput system
FRAXC: common fragile site on the X chromosome site-C
gRNA: guide RNA
H3K4me2: histone H3 dimethylated lysine 2
H3K9me3: histone H3 trimethylated at lysine 9
H3K27Ac: histone H3 acetylated at lysine 27
H3K27me3: histone H3 trimethylated at lysine 27
HMTase: histone metyltransferase
HP1β: heterochromatin protein 1 beta
IL1RAPL1: interleukin 1 receptor accessory protein-like 1
Kb: kilobase
KOs: knockouts
lncRNA: long noncoding RNA
LTR: long terminal repeat
Mb: megabase
qRT-PCR: quantitative reverse transcription PCR
RT-PCR: reverse transcription PCR
RNA-Seq: RNA sequencing
RPE1: retinal pigment epithelial cells
SETDB1: SET domain bifurcated 1
SNP: single nucleotide polymorphism
SMCHD1: structural maintenance of chromosomes flexible hinge domain containing 1
TALENs: transcription activator-like effector nuclease
Xa: active X chromosomes
XCI: X chromosome inactivation
Xi: inactive X chromosome
XIST: X inactive specific transcript
