# Supplementary figures and images for: Loss of SETDB1 decompacts the inactive X chromosome in part through reactivation of an enhancer in the IL1RAPL1 gene
Source: Epigenetics Chromatin. 2018 Aug 13;11:45. doi: 10.1186/s13072-018-0218-9 (PMC6088404; doi:10.1186/s13072-018-0218-9)

**a**

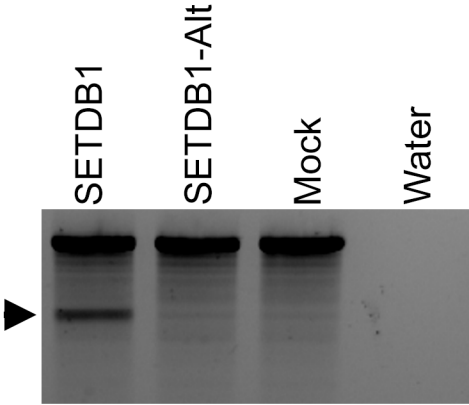

**b**

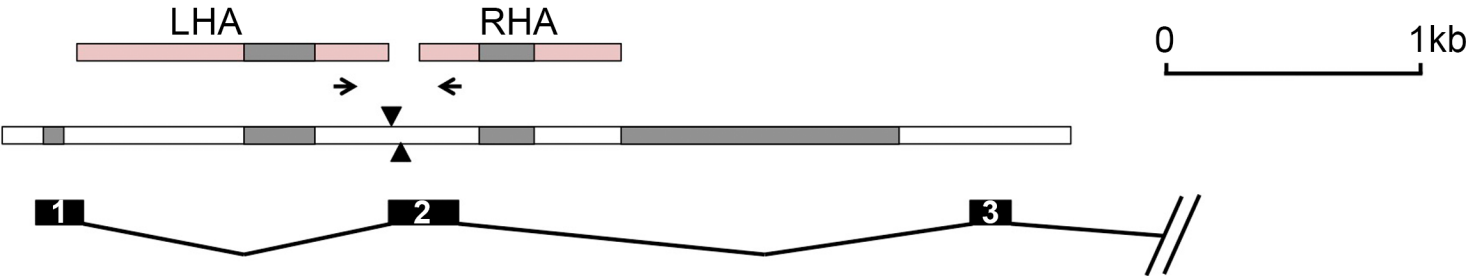

**c**

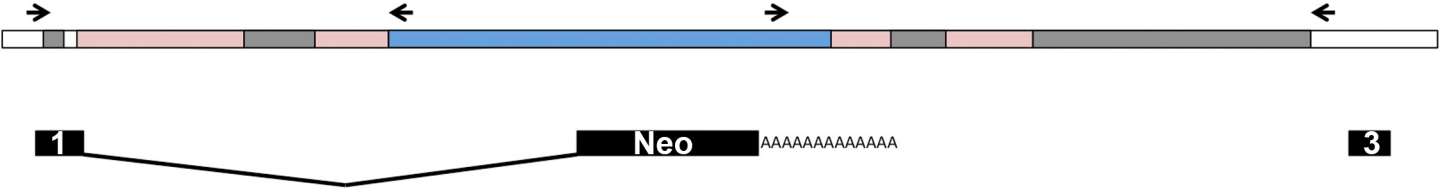

Supplement: Supplementary file 1 — Additional file 1. TALEN-assisted targeting of the SETDB1 gene. (a) Inverted ethidium bromide-stained agarose gel image showing the results of the Surveyor assay. 293 cells were transiently transfected with mammalian expression constructs expressing a previously reported active TALEN pair targeted to exon-2 of human SETDB1 [21] (SETDB1), the same TALEN pair assembled using an alternative platform [80] (SETDB1-Alt) and a transfection without DNA (Mock). Two days post-transfection, DNA was isolated from the cells and PCR performed across the cut site before performing the Surveyor assay. The right-facing black arrow head indicates the successful generation of Indels at exon-2 of SETDB1 in the SETDB1-TALEN transfection but not the alternate platform (SETDB1-Alt) or negative control mock sample. (b) Schematic representation showing the relative position of exons 1–3 of SETDB1. Gray-shaded intervals represent repetitive DNA sequences, whereas white regions are unique. The location of the left and right homology arms (LHA and RHA) are shown in pink above the genomic locus. Upward- and downward-facing black arrow heads indicate the binding location of the top-strand binding and bottom-strand binding TALENs. Inward-facing black arrows represent the location of oligonucleotide primers used for the Surveyor PCR. The location of exons 1–3 is shown as black boxes (exons) joined by black lines (introns) below the map. (c) Schematic representation of a successfully promoter trap targeted SETDB1 locus. The blue shaded interval represents the integrated pSEPT vector cloned between the LHA and RHA that contains the promoterless neomycin cassette. Inward-facing arrows show the approximate location of oligonucleotide primers used to amplify between the pSEPT vector and genomic locations up and downstream of the homology arms that are used for screening of clones by PCR. Beneath the map is a schematic showing the splicing of exon-1 to the neomycin cassette followed by transcription ter [file 13072_2018_218_MOESM1_ESM.pdf]

### Left Integrity Screen

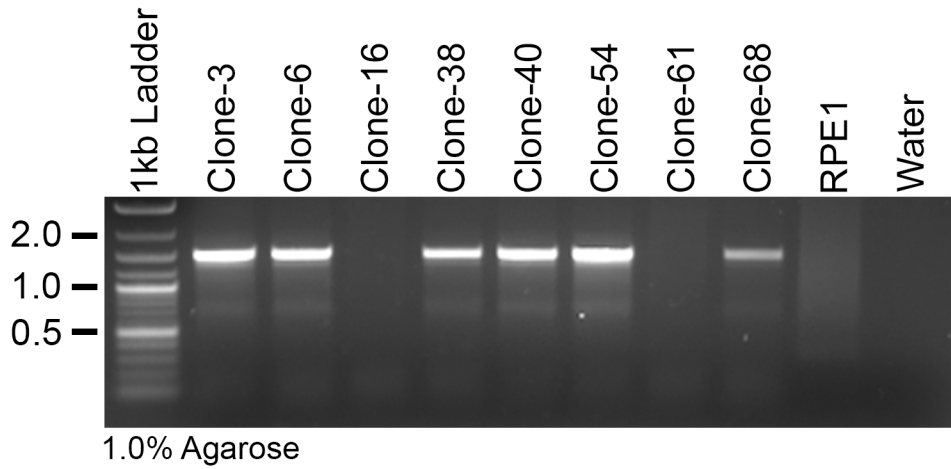

### TALEN Cut-Site Screen

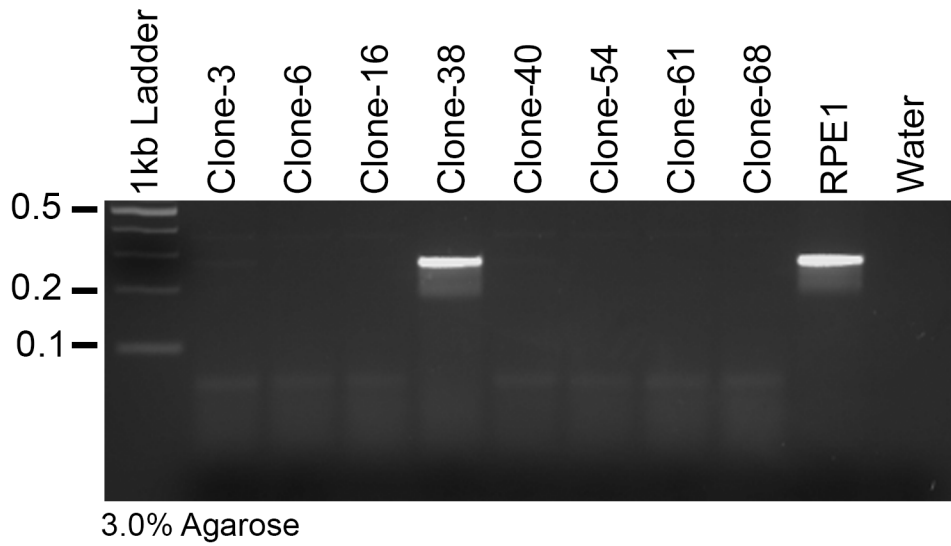

### Right Integrity Screen

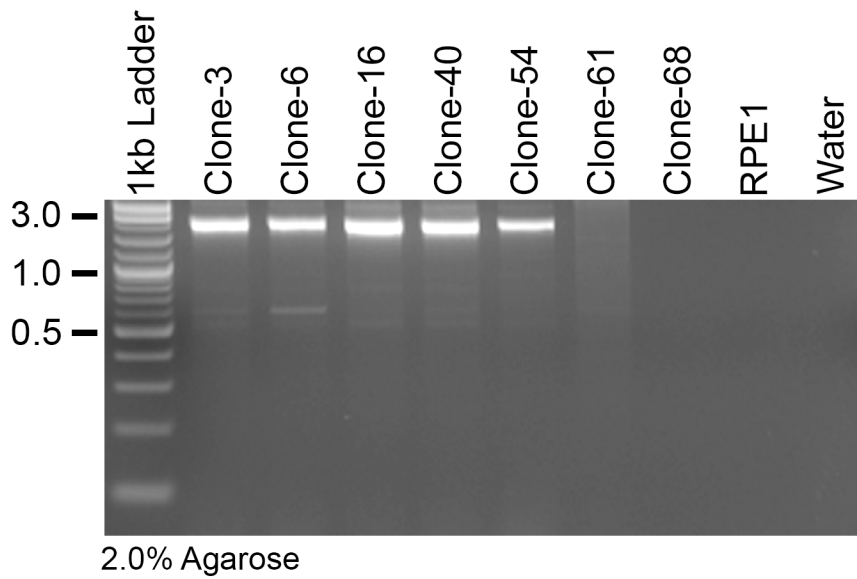

Supplement: Supplementary file 2 — Additional file 2. PCR screening for SETDB1-targeted clones. Representative examples of PCR screening results in searching for SETDB1 targeting. Each image shows an ethidium bromide-stained agarose gel with results for correct left homology arm integration (top), right homology arm integration (bottom) and the presence or absence of the TALEN cut site (middle). Molecular weight markers are in the first lane of each gel, and the sizes of fragments in kb are indicated to the left. PCR results for independent clones are shown in each lane and labeled above. Negative controls of RPE1 genomic DNA and water are shown at the far right of the gels. Successfully targeted clones generate a 1512 bp product for the left side and a 2183 bp product for the right side. The TALEN cut-site PCR generates a 269 bp product if the interval is intact or only targeted at one SETDB1 allele. Clones 3, 6 and 40 were selected for further analysis. All three are positive for the correct left and right targeting and are negative for the cut site PCR. [file 13072_2018_218_MOESM2_ESM.pdf]

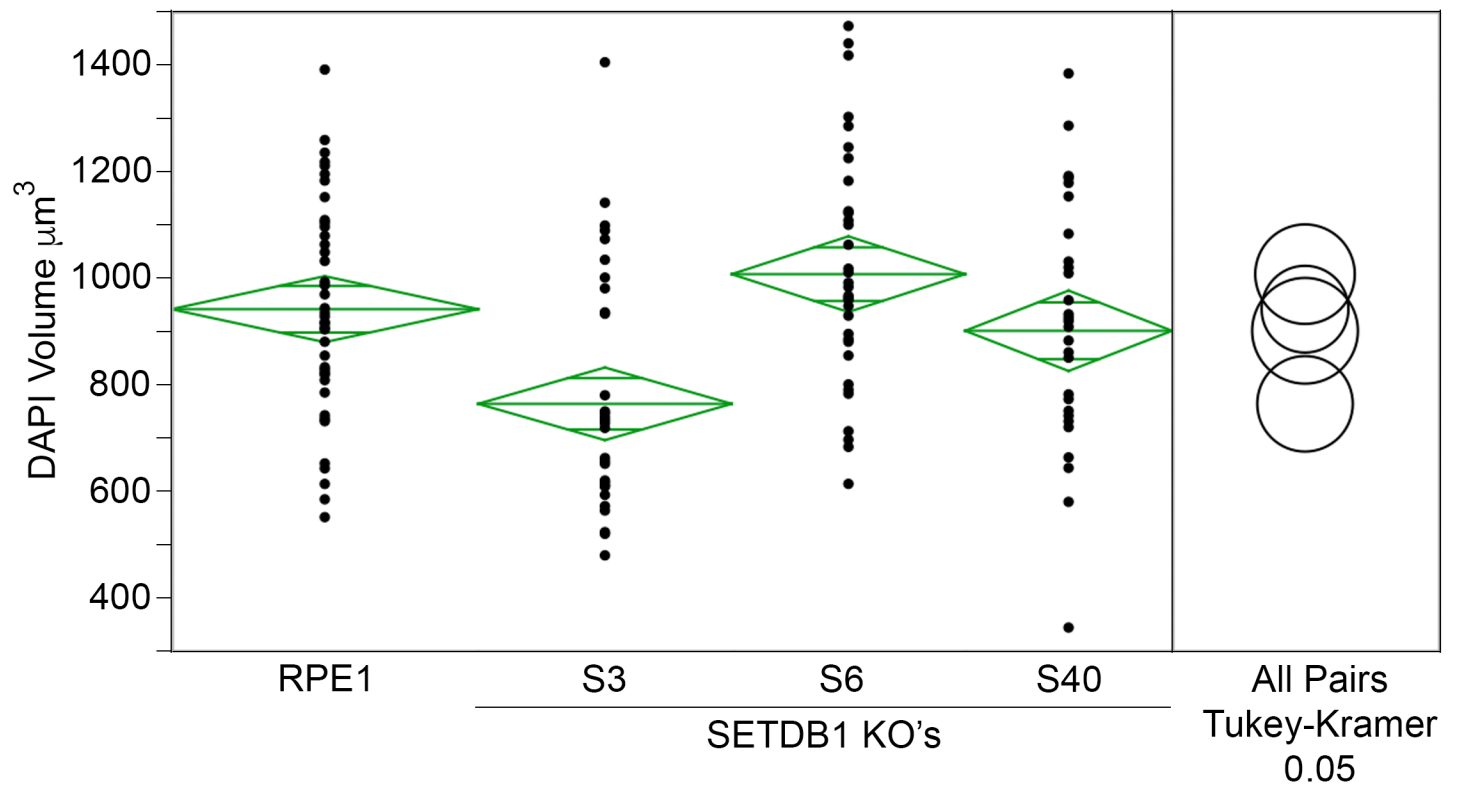

Supplement: Supplementary file 3 — Additional file 3. Nuclear volume measurements. Graph shows the results of nuclear volume measurements (based on DAPI volume) in RPE1 alongside the three SETDB1 mutants, S3, S6 and S40 (x-axis). DAPI volumes are indicated on the y-axis. Each black dot indicates a measurement made for individual nuclei in each sample. The green diamonds show the mean (central horizontal line) and 95% confidence interval between the apexes of the diamond. The width of the diamond is proportional to the sample size with wider diamonds indicating more measured nuclei. Circles on the far right show all pairs Tukey–Kramer 0.05 p value spread with significance presented by the angle of intersection between circles. None of the SETDB1 mutants have nuclear volumes that are significantly bigger than parental RPE1. [file 13072_2018_218_MOESM3_ESM.pdf]

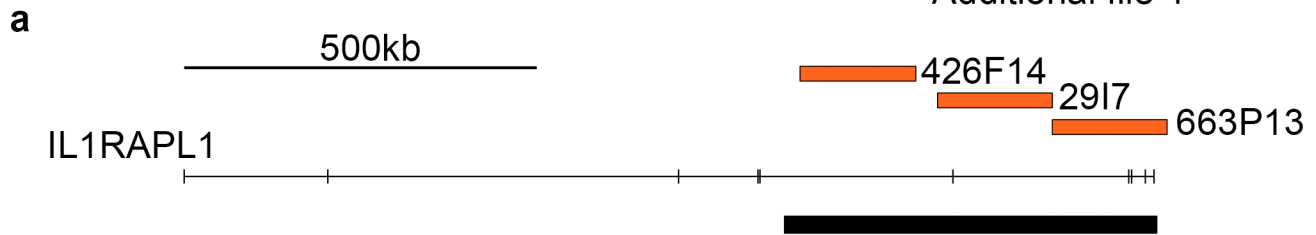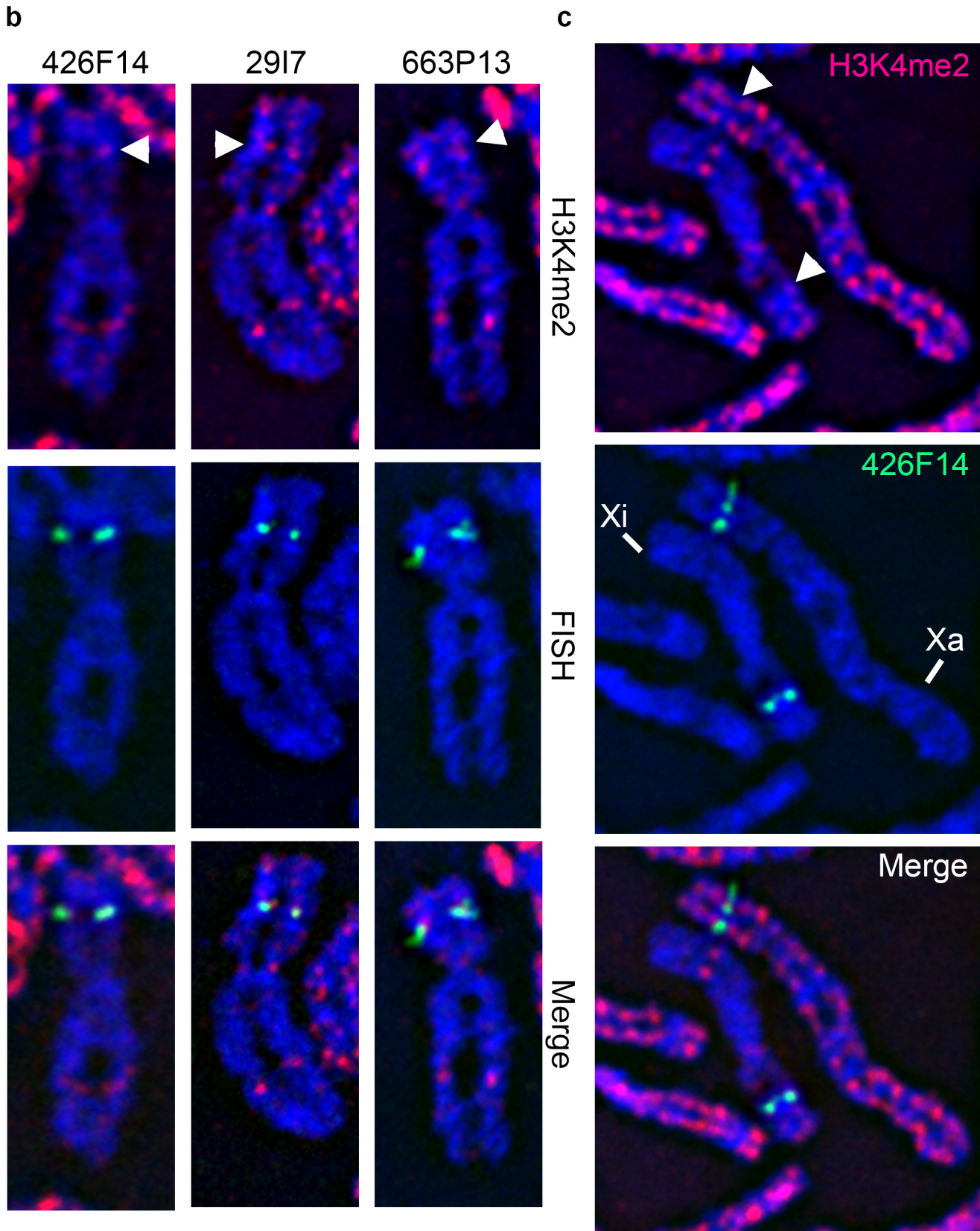

Supplement: Supplementary file 4 — Additional file 4. Validation of the SETDB1 mutant-specific H3K4me2 Xi band within the 3′ end of the IL1RAPL1 gene. (a) Schematic map of the IL1RAPL1 gene locus. The horizontal line represents introns bisected by vertical lines corresponding to exons. The solid black bar beneath the map indicates the approximate extent of chromatin change observed by ChIP-Seq (Fig. 2d). The orange bars represent the location of the indicated BAC clones. (b) Top panel shows representative examples of metaphase Xi in SETDB1 mutant S40 showing the distribution of H3K4me2 by indirect immunofluorescence (red) merged with DAPI (Blue). White arrow heads indicate the location of the novel H3K4me2 band observed in the mutant clones. The second row shows the hybridizing BAC probe signal (green) merged with DAPI (blue), whereas the last row shows a merge of all three. (c) Top panel shows an example of the H3K4me2 indirect immunofluorescence pattern (red) merged with DAPI (Blue) on the Xa alongside the Xi. The Xa-specific translocation of chromosome 10 at Xq28 results in a substantially longer Xa relative to the Xi, facilitating the ability to readily distinguish the two chromosomes. White arrow heads indicate the presence of the H3K4me2 band on the Xi but not Xa. The second panel shows the hybridization pattern for BAC probe 426F14 (Green) merged with DAPI (Blue). The bottom panels show a merge of all three. [file 13072_2018_218_MOESM4_ESM.pdf]

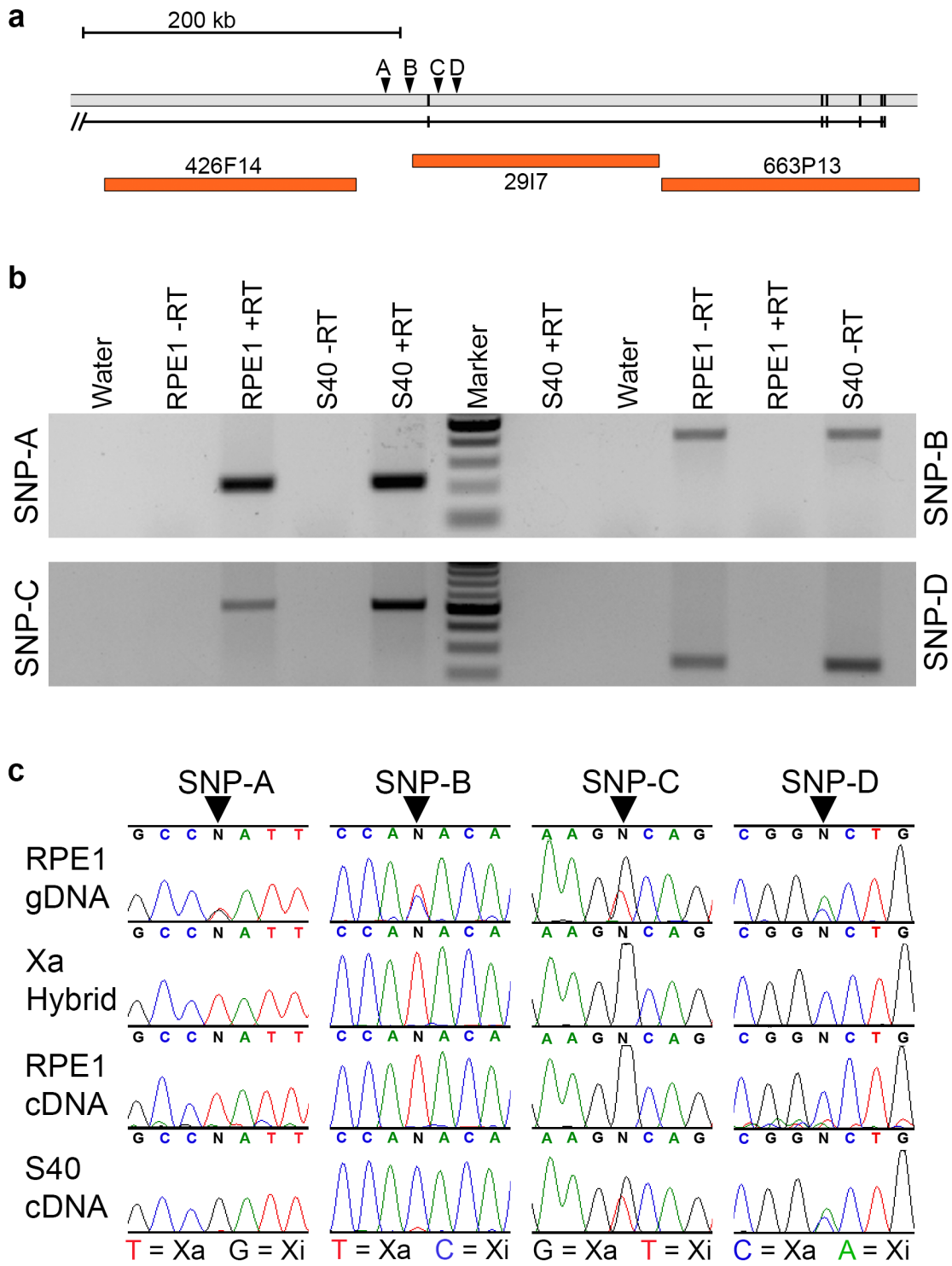

Supplement: Supplementary file 5 — Additional file 5. Genotyping results for transcripts originating from the 3′ end of IL1RAPL1. (a) Schematic map of the 3′ end of IL1RAPL1. The gray bar represents the genomic locus and black vertical lines indicate exons. The relative location of SNPs A–D is indicated above the genomic map. Below the genomic map is a representation of the IL1RAPL1 primary transcript followed by the relative position of BAC clones. (b) Inverted ethidium bromide-stained agarose gel image showing RT-PCR results with primers spanning SNPs A, B, C and D. Each gel shows RT-PCR results for a negative control water sample, cDNA prepared with reverse transcriptase (+RT) and without reverse transcriptase (− RT) for parental RPE1 and SETDB1 mutant S40. (c) DNA sequence traces for SNPs A, B, C and D in RPE1 genomic DNA, genomic DNA isolated from a somatic cell hybrid in which the RPE1 Xa is the only human chromosome present, and cDNA samples from RPE1 and SETDB1 mutant S40. The downward-facing black arrow heads at the top indicate the location of the SNP that appears as two merged traces in samples containing material from both alleles. [file 13072_2018_218_MOESM5_ESM.pdf]

**a**

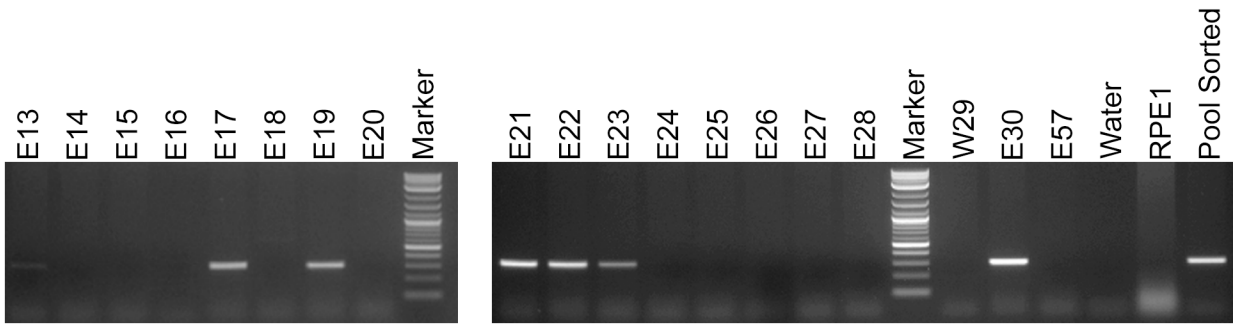

**b**

E21: IL1RAPL1 Enhancer  $\Delta$ Xa

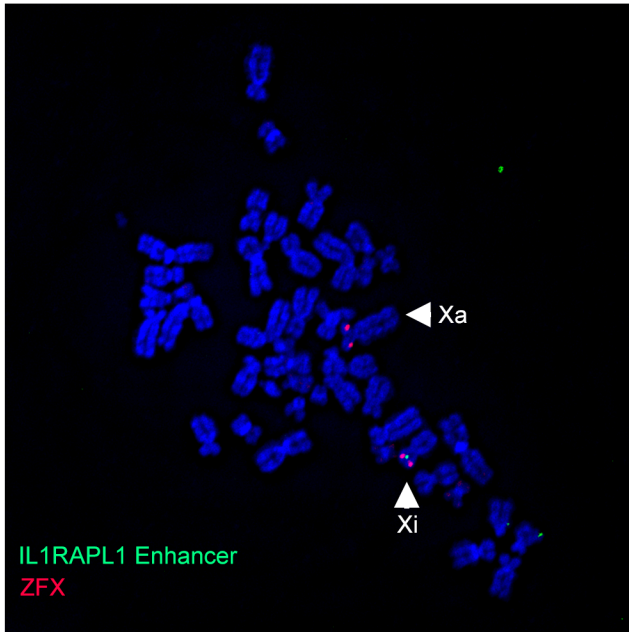

E30: IL1RAPL1 Enhancer  $\Delta$ Xi

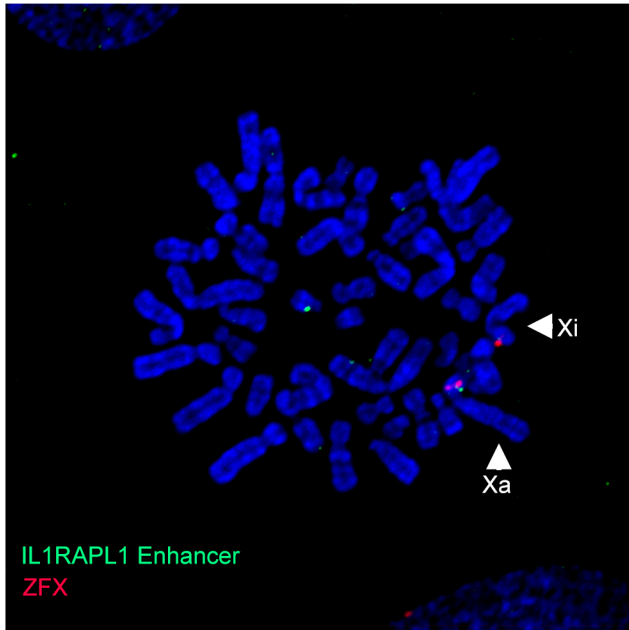

**c**

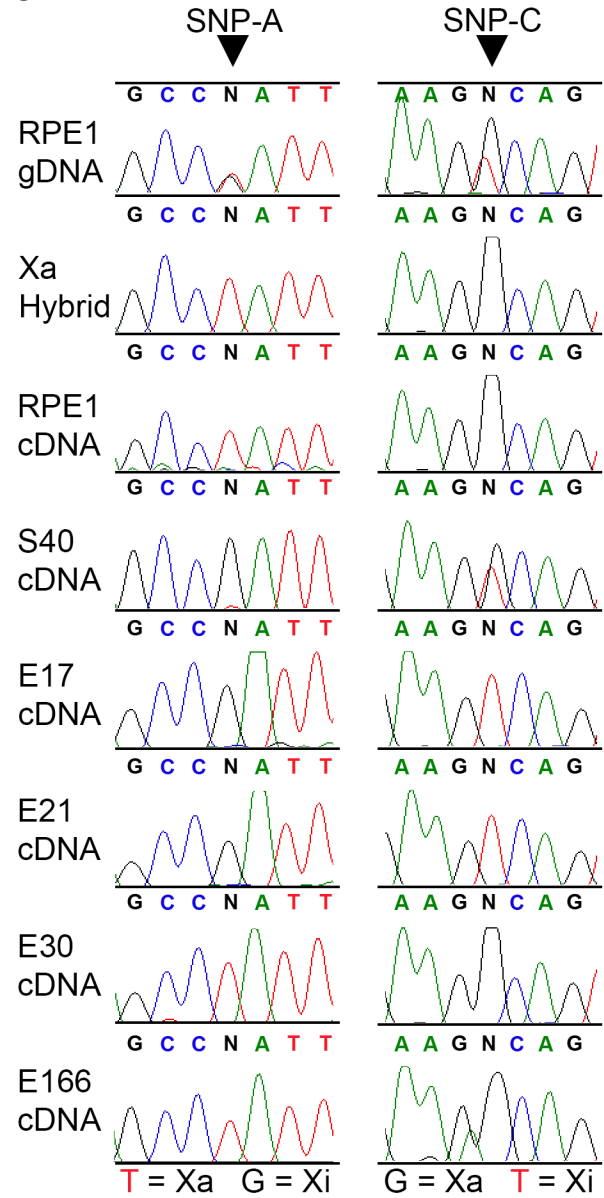

Supplement: Supplementary file 7 — Additional file 7. Validation of successful deletion of the IL1RAPL1 enhancer and the impact on allele expression by SNP genotyping. (a) Ethidium bromide-stained agarose gel images showing PCR results from individual clones exposed transiently to the gRNA pair flanking the enhancer locus. Successful deletion of the enhancer interval is indicated by the presence of a PCR product. Parental RPE1 and water are negative controls, whereas the “Pool Sorted” sample represents a pool of FACS sorted RPE1 cells exposed to the gRNAs as a positive control. (b) Dropped metaphase chromosome spreads for the indicated clones showing the hybridizing FISH signals for Xp control BAC probe ZFX (Red) and a probe generated from the enhancer deleted region (Green) merged with DAPI (Blue). The Xa and Xi are indicated. (c) DNA sequence traces for SNPs A and C in RPE1 genomic DNA, genomic DNA isolated from a somatic cell hybrid in which the RPE1 Xa is the only human chromosome present, and cDNA samples from RPE1, SETDB1 mutant S40 and four independent enhancer mutants. The downward-facing black arrow heads at the top indicate the location of the SNP that appears as two merged traces in samples containing material from both alleles. [file 13072_2018_218_MOESM7_ESM.pdf]
