# Supplementary material for: Loss of SETDB1 decompacts the inactive X chromosome in part through reactivation of an enhancer in the IL1RAPL1 gene
Source: Epigenetics Chromatin. 2018 Aug 13;11:45. doi: 10.1186/s13072-018-0218-9 (PMC6088404; doi:10.1186/s13072-018-0218-9)
Supplement: Supplementary file 6 — Additional file 6. List of oligonucleotides used in this study. All oligos were obtained from Eurofins Genomics LLC. Oligo names are given in column-1, the corresponding sequence is in column-2, and what the oligo was used for is listed in column-3. [file 13072_2018_218_MOESM6_ESM.pdf]

Additional file 6

List of oligonucleotides used in this study. All oligos were obtained from Eurofins Genomics LLC.

Oligo/Target	Sequence	Use
SETDB1-TAL-F	GTGTTAGCGTACAGCAGAAG	Surveyor assay
SETDB1-TAL-R	GTGAGCCACCTCAGATTCTT	Surveyor assay
SETDB1-LHA-Fwd	GCGGCCGCCCTTGAAGCTGGAAGACG	Left homology arm
SETDB1-LHA-Rev	TCTAGATTGTCCTCTGCATGGAACAG	Left homology arm
SETDB1-RHA-Fwd	GAATTCTCTATGGAGGAACTTCGGC	Right homology arm
SETDB1-RHA-Rev	GCGGCCGCAGTGACAACTAGAGCACCAG	Right homology arm
pSEPT-R2	CTGACAATCTTAGCGCAGAAG	Left homology arm screen
SETDB1-Fwd2	GCACTAAAGGTTTGCTTCCG	Left homology arm screen
pSEPT-F2	AACCACAACTAGAATGCAGTG	Right homology arm screen
SETDB1-R2	GGTATTGTAGTCCCAGCTTG	Right homology arm screen & SETDB1 Exon 1-3 RT-PCR
SETDB1-Flox-F	ATAACAGGCAGCAGAGTAGG	Cut-site integrity screen
SETDB1-Flox-R	TCTTCTCCAGTTCCTCATCG	Cut-site integrity screen
SETDB1-Fwd	TGAGTTGTGAGTCTGGGGTC	SETDB1 Exon 1-3 RT-PCR
SETDB1-RT-F2	CCGAAGACTCATGGCTTCTG	SETDB1 Exon 18-21 RT-PCR
SETDB1-RT-R2	GATCATGGGTATCCACGAAG	SETDB1 Exon 18-21 RT-PCR
SETDB1-RT-F1	ACATCCTCAGCCTCTGCACT	SETDB1 qRT-PCR
SETDB1-RT-R1	TTCCAGTACCGGTCAGATCC	SETDB1 qRT-PCR
IL1RAPL1-5’	PrimePCR PreAmp (Bio-Rad 10041595)	5’ qRT-PCR
IL1RAPL1-3’	RT^2 qPCR Primer Assay (Qiagen PPH01679A)	3’ qRT-PCR
GAPDH-Fwd	GAAGGTGAAGGTCGGAGTC	RT-PCR
GAPDH-Rev	GAAGATGGTGATGGGATTTC	RT-PCR
qGAPDH-Fwd-2	TGCACCACCAACTGCTTAGC	GAPDH qRT-PCR
qGAPDH-Rev-2	GGCATGGACTGTGGTCATGAG	GAPDH qRT-PCR
IL1-SNP-2965-F	CCAAGGTTCAAACCACATCTG	SNP-A PCR (rs140261831)
IL1-SNP-2965-R	TACAGCTGGTGGAGATAGAG	SNP-A PCR (rs140261831)
IL1-SNP-2967-F	TCTGGCTTCTGTCCTCACTG	SNP-B PCR (rs113985890)
IL1-SNP-2967-R	TGTTGGCTAGAACTCTCAGC	SNP-B PCR (rs113985890)
IL1-SNP-2969-F	AGCCCTAGTTCTAGGACAGG	SNP-C PCR (rs190632360)
IL1-SNP-2969-R	GTACTAGCAGCTGGGTCAC	SNP-C PCR (rs190632360)
IL1-SNP-2970-F	GACATTTACCAGGTGTGAGG	SNP-D PCR (rs144991617)
IL1-SNP-2970-R	ATAACATGCTCACCTTGTGAC	SNP-D PCR (rs144991617)
IL1RAPL1-Enh-1T	CACCGTGAGTCTTGGTGCATCAAAC	Enhancer gRNA adapter
IL1RAPL1-Enh-1B	AAACGTTTGATGCACCAAGACTCAC	Enhancer gRNA adapter
IL1RAPL1-Enh-4T	CACCGTAGACCATGACTAAGCCAAC	Enhancer gRNA adapter
lL1RAPL1-Enh-4B	AAACGTTGGCTTAGTCATGGTCTAC	Enhancer gRNA adapter
IL1RAPL1-Enh-F1	GCAATCAGTTGGAATTCATCC	Enhancer deletion screen
lL1RAPL1-Enh-R1	TTTCTGCACTGGCAAAGGTG	Enhancer deletion screen
IL1RAPL1-Enh-R2	AGGAAACCGTGGTCAACATG	gRNA site integrity (w/IL1RAPL1-Enh-F1)
IL1RAPL1-Enh-F2	GCAGAAGCTTTGTCAACTGAG	gRNA site integrity (w/IL1RAPL1-Enh-R1)
IL1RAPL1-En-F1	GTCGACATGAGTCATCAGTCACTGCC	Large enhancer fragment
IL1RAPL1-En-R1	GGATCCAAAGTGCTGGGATTACAGGC	Large enhancer fragment
IL1RAPL1-En-F3	GTCTCTATCTCTTTAGCTGC	Sequencing enhancer fragment
IL1RAPL1-En-F2	GTCGACCAGTTGCTTAGTCACTCCTC	Small enhancer fragment
IL1RAPL1-En-R2	GGATCCTGCAAGGCAATGTACAGGAC	Small enhancer fragment
IL1RAPL1-ERVL-F	TACCTCAGCCTCCCAAGTAG	New promoter fragment
IL1RAPL1-ERVL-R	CTGCAAATGATCTACCTGAC	New promoter fragment
