# Supplementary material for: Loss of SETDB1 decompacts the inactive X chromosome in part through reactivation of an enhancer in the IL1RAPL1 gene
Source: Epigenetics Chromatin. 2018 Aug 13;11:45. doi: 10.1186/s13072-018-0218-9 (PMC6088404; doi:10.1186/s13072-018-0218-9)
Supplement: Supplementary file 8 — Additional file 8. List of BAC clones used in this study. BAC clone names are given in column-1 and the corresponding gene content is listed in column-2 [file 13072_2018_218_MOESM8_ESM.pdf]

**Additional file 8**
List of BAC clones used in this study.

| BAC Clone | Content |
|---|---|
| RP11-1107D4 | Contains ZFX gene |
| RP11-667E3 | 5’ end of IL1RAPL1 |
| RP11-187F12 | 5’ end of IL1RAPL1 |
| RP11-426F14 | 3’ end of IL1RAPL1 |
| RP11-29I7 | 3’ end of IL1RAPL1 |
| RP11-663P13 | 3’ end of IL1RAPL1 |
