# Supplementary material for: Loss of SETDB1 decompacts the inactive X chromosome in part through reactivation of an enhancer in the IL1RAPL1 gene
Source: Epigenetics Chromatin. 2018 Aug 13;11:45. doi: 10.1186/s13072-018-0218-9 (PMC6088404; doi:10.1186/s13072-018-0218-9)
Supplement: Supplementary file 9 — Additional file 9. IL1RAPL1 RNA FISH data for the BACs indicated in the cells listed. Xi expression is defined as a single associated with the XIST RNA cloud. Each column represents data from replicate experiments. Therefore, a clone with BAC data in two columns indicates that the experiment was done twice, whereas that in four columns indicates the experiment was done on four occasions [file 13072_2018_218_MOESM9_ESM.pdf]

Additional file 9

IL1RAPL1 RNA FISH data for the BACs indicated in the cells listed.

Cells	Expression site	BAC-667E3				BAC-187F12			
RPE1	Xa	26	28	38	20	28	20	22	16
	Xi	0	2	0	0	2	0	2	0
	Xa & Xi	2	0	4	0	0	0	0	0
	None	72	70	58	80	70	80	76	84
S3	Xa	18	8			12	8		
	Xi	0	2			2	0		
	Xa & Xi	0	0			0	0		
	None	82	90			86	92		
S6	Xa	16	10			16	28		
	Xi	0	0			0	2		
	Xa & Xi	2	2			0	4		
	None	82	88			84	66		
S40	Xa	10	6	22		10	8	14	
	Xi	2	0	2		0	2	0	
	Xa & Xi	0	2	2		0	0	2	
	None	88	92	74		90	90	84	

Cells	Expression site	BAC-426F14			BAC-29I7		
RPE1	Xa	10	14		12	24	
	Xi	0	6		0	4	
	Xa & Xi	0	0		0	0	
	None	90	80		88	72	
S3	Xa	0	0		6	10	
	Xi	50	42		30	16	
	Xa & Xi	50	56		62	72	
	None	0	2		2	2	
S6	Xa	0	4		0	12	
	Xi	40	56		26	26	
	Xa & Xi	52	16		72	60	
	None	8	24		2	2	
S40	Xa	2	2	2	26	2	8
	Xi	64	42	60	22	40	4
	Xa & Xi	34	52	38	30	40	88
	None	0	4	0	22	18	0

Cells	Expression site	BAC-663P13			
RPE1	Xa	12	4		
	Xi	0	0		
	Xa & Xi	0	0		
	None	88	96		
S3	Xa	6	8		
	Xi	0	4		
	Xa & Xi	94	88		
	None	0	0		
S6	Xa	10	12		
	Xi	12	8		
	Xa & Xi	76	80		
	None	2	0		
S40	Xa	20	20	8	2
	Xi	10	6	4	10
	Xa & Xi	70	72	88	84
	None	0	2	0	4
